# Comparison of protons and very high-energy electrons transmission pencil-beam-scanning for FLASH radiotherapy

**DOI:** 10.1016/j.phro.2025.100860

**Published:** 2025-11-01

**Authors:** Flavia Gesualdi, Louis Ermeneux, Pierre Lansonneur, Mateusz Sitarz, Pierre Loap, Gilles Créhange, Anthony Magliari, Ludovic De Marzi

**Affiliations:** a Institut Curie – Proton Therapy Center of Orsay, Radiation Oncology Department, 15 Rue Georges Clémenceau, 91400 Orsay, France; b Institut Curie, PSL Research University, University Paris Saclay, INSERM U1288 LITO, Campus universitaire, Orsay 91898, France; c Varian Medical Systems Inc., 3100 Hansen Way, Palo Alto 94304, USA

**Keywords:** FLASH, VHEE, Transmission protons, Shoot-through protons, Ultra-high dose rates

## Abstract

**Background and purpose:**

Very High-Energy Electron (VHEE) radiotherapy stands as a promising alternative to proton therapy in view of the FLASH effect, which allows for differential sparing of healthy tissues under ultra-high dose rate irradiation. This study compared the quality of transmission Pencil-Beam-Scanning proton and VHEE treatment plans, with emphasis on dose rate quantification relevant to the assessment of the FLASH effect.

**Materials and methods:**

Proton (250 MeV) and VHEE (200 MeV and 150 MeV) treatment plans were designed for four patient cases. Plans were optimized using an in-house developed spot weight and position optimization algorithm. Objectives were set through scorecards based on RTOG protocols. Dose rate estimations were based on beam parameters of the ProBeam system for protons and of a realistic VHEE system. For each structure, a new, model-free FLASH index was calculated.

**Results:**

VHEE treatment plans achieved a quality comparable to that of protons. While the conformity and homogeneity were similar, FLASH indices tended to be higher for protons due to higher dose rates covering more healthy tissues. A pulse repetition frequency of 500 Hz was found necessary to attain FLASH-compatible dose rates (≥40 Gy/s).

**Conclusions:**

The importance of treatment parameters (such as pulse repetition frequency) for VHEEs to reach ultra-high dose rates was assessed. With their high plan quality, VHEEs could constitute a viable alternative to proton transmission plans and a promising modality for FLASH therapy. The proposed FLASH index offers a robust tool to compare the FLASH potential across treatment modalities.

## Highlights

• Transmission pencil beam scanning with protons and electrons achieved similar quality.
• A new model-independent FLASH index quantified the treatment plan FLASH potential.
• Proton plans reached higher FLASH indices due to larger ultra-high-dose rate coverage.
• Electron beams needed ≥500 Hz, ideally 1000 Hz, to reach proton dose rates.

## Graphical abstract

**Figure:**
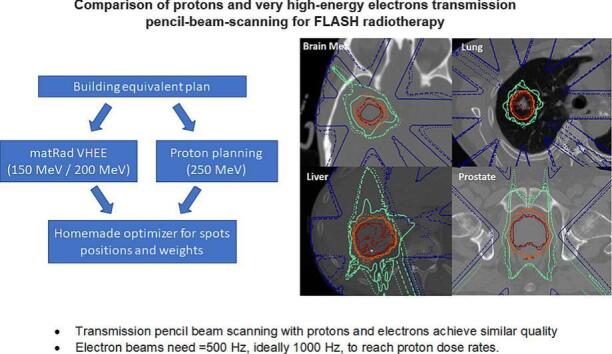

## 1 Introduction

The FLASH effect refers to the phenomenon in which dose delivered at ultra-high dose rates (UHDRs) allows for an increased healthy tissue sparing while maintaining anti-tumor efficacy [1]. First discovered a decade ago [2,3], FLASH was recently clinically demonstrated for proton therapy [4]. The mechanism behind the FLASH effect is still to be understood [5], but experimental evidence suggests that dose of at least 8–10 Gy [6] delivered at 40–100 Gy/s may be needed [1,7].

Current proton machines are already capable of reaching the necessary UHDRs for FLASH [8]. Standard intensity modulated proton therapy (IMPT) is however incompatible with FLASH as the energy-layer shifting time is typically too large (∼200–1000 ms). Therefore, either transmission beams or a single energy layer combined with range modulators are necessary to remain at UHDRs [1]. The highest dose rates can then be reached at the highest energy, at the cost of a reduced dose conformity.

Construction and operation of PT centers translates into a high cost per treatment and a low accessibility [9]. Very-High Energy Electron (VHEE) radiotherapy in the energy range of 100 MeV to 250 MeV are capable of reaching deep-seated tumors (depth >10 cm) [10], constitutes a potential alternative to PT. While detailed comparison has never been conducted so far, VHEE could potentially be less expensive than proton, as being lighter particles of lower magnetic rigidity, they would thus need smaller magnets and shorter acceleration section [11]. VHEE beams could also be focused into a deep spread-out electron peak [12,13] and are minimally affected by tissue heterogeneities compared to other charged particle therapies, presumably due to low scattering and a smooth depth-dose profile [14]. Currently, non-clinical VHEE machines exist [[15], [16], [17]], while not having necessarily been designed or tuned to reach the UHDRs needed for FLASH, several VHEE accelerators are under development in this aim [[17], [18], [19], [20], [21], [22]].

Previous studies showed that FLASH-delivered transmission proton plans can have a comparable quality with respect to conventional IMPT on specific patient cases (e.g. lung/brain tumors) [23,24]. Shoot-through proton beams can also lower linear energy transfer distributions in organs at risk (OARs) and be robust against density variations [23,24]. Transmission proton and VHEE plans can also offer a similar plan quality [25]. Several clinical trials are underway with FLASH transmission beams (for extremity bones or lung metastases) [4,26]. For these reasons, we have decided to consider only transmission beams in this study.

In this work, we aim to compare proton and VHEE plans for several representative clinical cases, making a special emphasis on dose rate quantification and on the assessment of their UHDR delivery capability. To our knowledge, this is the first study to do so.

## 2 Materials and methods

### 2.1 Treatment planning and optimization

Proton and VHEE PBS transmission treatment plans were designed and reviewed by a clinician for four patient cases, covering different tumor locations and sizes: 1) Brain 2) Lung 3) Liver 4) Prostate. Patient imaging data were anonymized and used in accordance with institutional ethical guidelines, approval and informed consent obtained for research use. Table S1 in supplementary material provides the plan characteristics of the four cases. The beam energy for the proton plans was of 250 MeV, which is the highest energy delivered by the ProBeam system [27]. For VHEE energies, 150 MeV and 200 MeV were selected due to their clinically desirable properties for shoot-through beam treatments [28] and to align with performance of future FLASH-compatible VHEE accelerators (inferred from current systems).

Treatment plan optimization was performed through an in-house Python-based spot weight and position optimization algorithm [29]. To reduce computational cost, the algorithm uses precomputed dose-influence matrices on a 2 mm spot grid for each field. The dose influence of shifted spot positions is obtained through interpolation on the precomputed grid. Validation with a finer 1 mm grid showed numerically identical results. The dose influence matrices were calculated using Eclipse™ for protons, while, for VHEE, a recent implementation of a VHEE model [30] in matRad [31] was utilized. The latter is based on an analytical model extensively validated against Monte Carlo simulations. Spots initial positions were configured following a centroidal Voronoi tessellation inside the PTV, and laying spots at its border, approximately every ∼ 5 mm [32]. This spacing was selected to achieve a balance between minimizing the number of scanned spots, while preserving dose homogeneity in target. For VHEE, spots are described using a double-Gaussian parametrization, with a size (sigma) of 5 mm following Refs. [30,33], comparable to those of protons of approximately 10 mm (FWHM) [34]. The optimization objectives were set through scorecards [35] based on RTOG protocols [[36], [37], [38], [39]] to ensure compliance with validated clinical criteria. These objectives were the exact same for both proton and VHEE plans of a given patient, they can be found in Tables S2–S5 in the Supplementary Material.

Plans quality were evaluated from the comparison of the distribution of the overall dose, of the dose delivered at a dose rate ≥40 Gy/s, of the dose-volume histograms (DVHs), and of the plan metrics. For the PTV, the computed plan metrics were the homogeneity index $HI_{98\%}=D_{98\%}/D_{2\%}$, the conformity indices $CI_{95\%}$ and $CI_{50\%}$, calculated as $CI_{X\%}=V_{\mathrm{PTV},X\%}/V_{\mathrm{tot},X\%}$ [40,41], and the dose quantiles $D_{2\%}$, $D_{95\%}$, and $D_{98\%}$. For the OARs, the computed metrics were the $D_{2\%}$, $D_{\mathrm{mean}}$, the percentage of the volume receiving more than 10 Gy, and the FLASH index defined in Sec. 2.2.

### 2.2 Dose rate and FLASH quantification

For protons, the computation of dose rates was based on the characteristics of the existing ProBeam system [27]. For VHEE, the parameters were extracted from a previously published preliminary design of linac for FLASH radiotherapy [22]. Pulse repetition frequency (PRF) values between 100 and 1000 Hz were considered, corresponding to the orders of magnitude found in clinical linacs [[42], [43], [44], [45]]. It was further assumed that the time taken to deflect the electron beam was shorter than the time between pulses, a feature that magnets of current proton accelerators could easily achieve with electrons thanks to their lower magnetic rigidity [46].

**Fig. 1:**
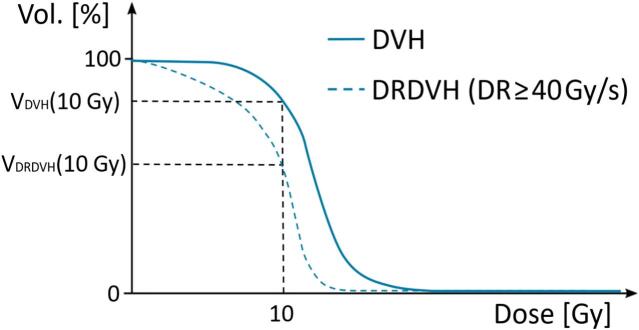
Illustration of a cumulative dose-volume histogram (DVH, solid blue line) and of a dose-rate dose volume histogram (DRDVH, dashed blue line). (For interpretation of the references to colour in this figure legend, the reader is referred to the web version of this article.)

For each field, the dose rate was mapped using an in-house developed MATLAB code, using a previously published PBS dose rate definition [47].^2^ A second dose map was calculated, consisting of the sum over the fields and fractions of the dose delivered at a PBS dose rate ≥40 Gy/s (necessary to achieve the FLASH effect). Fig. 1 shows an illustration of cumulative dose-volume histograms of the total dose (DVH) and of the dose delivered at a PBS dose rate ≥40 Gy/s (referred to as dose-rate dose-volume histogram, DRDVH).

Assessing the proportion of a given structure likely to benefit from the FLASH effect by fulfilling the needed UHDR conditions is of key interest. For that purpose we have introduced a FLASH index metric, $FI_{\left(XGy,XGy/s\right)}$, calculated for each structure of each treatment plan, as the fraction of voxels receiving dose > X Gy at a dose rate ≥ X Gy/s, $\mathrm{Vo}l_{\mathrm{DRDVH}}\left(XGy,XGy/s\right)$, divided by the fraction of voxels of the structure receiving dose > X Gy, $\mathrm{Vo}l_{\mathrm{DVH}}\left(XGy\right)$ [48]:

(1) $$FI_{\left(10Gy,40Gy/s\right)}=\frac{\mathrm{Vo}l_{\mathrm{DRDVH}}\left(XGy,XGy/s\right)}{\mathrm{Vo}l_{\mathrm{DVH}}\left(XGy\right)}$$

The FLASH index can be interpreted from Fig. 1 as the ratios of the cumulative DVHs at 10 Gy. An $FI_{\left(10Gy,40Gy/s\right)}$ value of 1 (respectively 0) means that all voxels that receive a dose >10 Gy are irradiated at a dose rate ≥40 Gy/s (resp. <40 Gy/s). If no voxels receive dose >10 Gy ($\mathrm{Vo}l_{\mathrm{DVH}}\left(10Gy\right)=0\%$), $FI_{\left(10Gy,40Gy/s\right)}$ tends to zero since $\mathrm{Vo}l_{\mathrm{DRDVH}}\left(10Gy,40Gy/s\right)\leq Vol_{\mathrm{DVH}}\left(10Gy\right)$. The FLASH index here defined can be understood as a plan quality metric associated with a certain OAR or region of interest, that can be looked at for several dose and dose-rate threshold combination. It reflects the proportion of the volume that received dose > X Gy under UHDR conditions, while remaining agnostic to the extent of the differential radiobiological effect induced by FLASH (which is not well modelled to date).

FLASH effect was reported in several previously published studies, to occur above a certain minimal dose and dose rate [6,7,49,50]. We decided to adopt a dose threshold of 10 Gy and show the results for a dose rate threshold of 40 Gy/s in the main manuscript. FLASH indices for several combination of dose threshold and/or a dose rate threshold were also calculated and included in the Supplementary Material to conduct a robustness evaluation.

## 3 Results

**Fig. 2:**
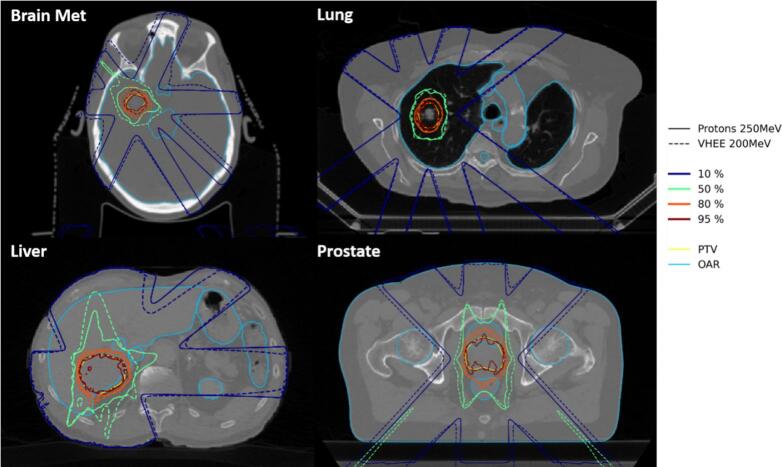
Isodose curves (relative to the prescribed dose, 10 % in blue, 50 % in green, 80 % in orange, and 95 % in red) for the brain metastasis (upper left), lung (upper right), liver (lower left), and prostate (lower right) cases. The proton 250 MeV plans are shown in solid lines and the VHEE 200 MeV plans in dashed lines. (For interpretation of the references to colour in this figure legend, the reader is referred to the web version of this article.)

The comparison of the dose distributions relative to the prescribed dose for the brain metastasis, lung, liver, and prostate cases, for the proton 250 MeV (solid lines) and VHEE 200 MeV (dashed lines) treatment plans led to similar dose distributions (Fig. 2). Same tendency was observed between the two VHEE 200 MeV and VHEE 150 MeV treatment plans (Fig. S1). It is worth highlighting that VHEE beams were capable of reaching all the studied deep-seated tumors. This could be seen in the PTV characteristic indexes such as CI_95_, which varied by a few percent between the two energies (Tables S6–S9).

**Fig. 3:**
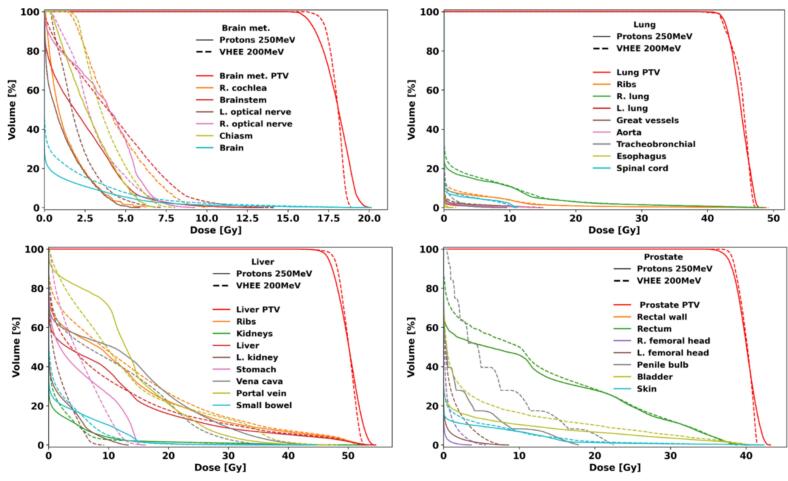
DVH comparison for the brain metastasis (upper left), lung (upper right), liver (lower left), and prostate (lower right) cases. The proton 250 MeV plans are shown in solid lines and the VHEE 200 MeV plans in dashed lines. Different colors identify the different OARs, while the PTV is for all cases in red. Abbreviations: “R.” for right, “L.” for left. (For interpretation of the references to colour in this figure legend, the reader is referred to the web version of this article.)

Plan quality evaluation conducted for every cases shows an equivalent target coverage between investigated modalities (Figs. 3, S2 and S3 and Tables S6–S9). Regarding the OARs, several DVHs fell lower with the proton plans, as confirmed by the values for D_2_ or D_mean_ (see Tables S6–S9 in the Supplementary Material). A detailed analysis of dosimetric indices for the various cases is provided in the Supplementary Material.

**Fig. 4:**
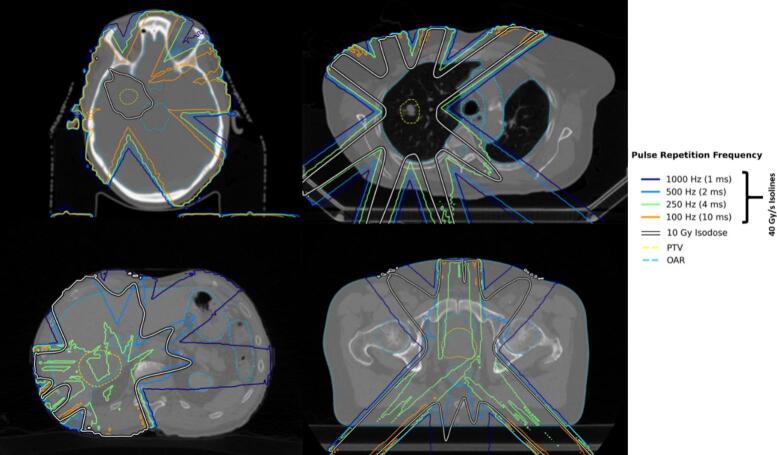
Comparison of the 10 Gy isodose curves (white lines with black border), and the isolines for dose rate ≥40 Gy/s for four different pulse repetition frequencies (solid color lines), considering VHEEs at 200 MeV, for the brain metastasis (upper left), lung (upper right), liver (lower left), and prostate (lower right) cases. The PTV and OARs are shown in yellow and light blue dashed lines respectively. (For interpretation of the references to colour in this figure legend, the reader is referred to the web version of this article.)

Figs. 4 and S4 showed that a PRF of 1000 Hz fully covered the 10 Gy isodose volume for all cases. PRF of 500 Hz covered all the 10 Gy isodose volumes for the brain metastasis and lung cases, but not in the prostate and liver cases, in which the DR ≥40 Gy/s isodose curves (color lines) did not fully contained the 10 Gy isodose curves. Lower PRF values poorly covered the 10 Gy isodose region in all cases except the brain one, which was well covered with all the evaluated PRFs, even at 100 Hz, and except for the lung where the 250 Hz PRF offered a satisfying coverage.

**Fig. 5:**
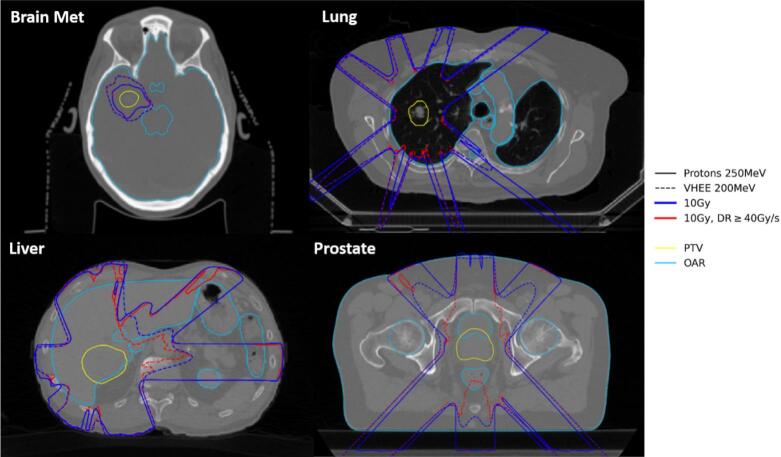
10 Gy isodose contour (blue) against the 10 Gy isodose contour delivered at a dose rate ≥40 Gy/s (red), for each patient case, for the proton 250 MeV (solid lines) and VHEE 200 MeV (dashed lines) treatment plans. The pulse repetition frequency of the VHEE 200 MeV plan was 500 Hz. (For interpretation of the references to colour in this figure legend, the reader is referred to the web version of this article.)

For the brain metastasis and lung cases (with the smaller PTV volumes) the 10 Gy isodose contours (in blue) and the 10 Gy, DR ≥40 Gy/s isodose contours (in red) overlapped almost completely (Fig. 5). The differences were between irradiation modalities (depicted with different line types), most notably in the brain case, where the 10 Gy and 10 Gy, DR ≥40 Gy/s isodose contours of the VHEE plan covered a larger area than the proton contours. Regarding the liver and prostate cases (which had larger PTV volumes), for the proton plans, the 10 Gy isodose contours and 10 Gy, DR >40 Gy/s isodose contours mostly overlapped. In these cases, VHEE performed worse than protons as the 10 Gy, DR >40 Gy/s regions were significantly smaller than the 10 Gy isodose regions. Differences between the VHEE energies of 150 MeV and 200 MeV were found to be small (Fig. S3 of Supplementary Material).

**Fig. 6:**
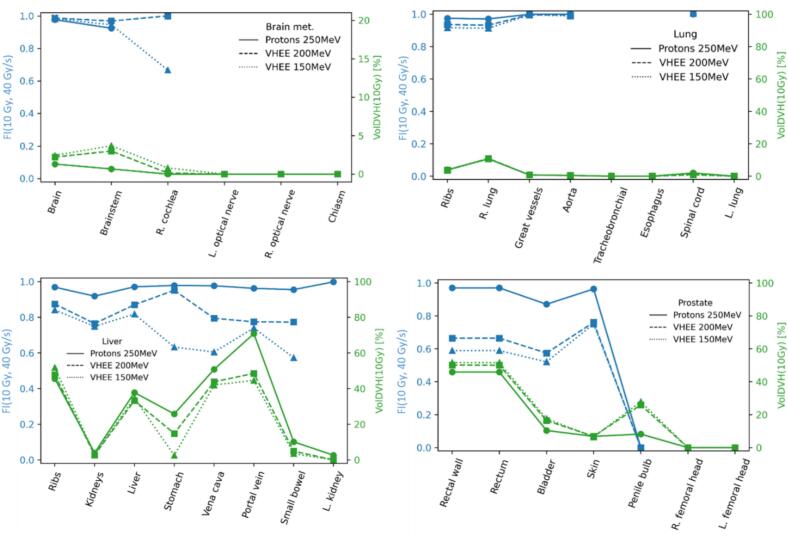
FLASH index $FI_{\left(10Gy,40Gy/s\right)}$ (c.f. Eq. (1), blue, left y-axis), and volume irradiated above 10 Gy, $\mathrm{Vo}l_{\mathrm{DVH}}\left(10Gy\right)$ (green, right y-axis), for the brain metastasis (upper left), lung (upper right), liver (lower left), and prostate (lower right) cases. Proton 250 MeV plans are shown in solid lines, the VHEE 200 MeV in dashed lines, and the VHEE 150 MeV in dotted lines. (For interpretation of the references to colour in this figure legend, the reader is referred to the web version of this article.)

The FLASH index values $FI_{\left(10Gy,40Gy/s\right)}$, were systematically smaller for the VHEE plans with respect to the proton plans (Fig. 6). Wherever $\mathrm{Vo}l_{\mathrm{DVH}}\left(10Gy\right)=0\;\%$, no associated $FI_{\left(10Gy,40Gy/s\right)}$ point was plotted (for example, for most brain OARs, and for some OARs for the lung and liver cases with the VHEE plans). The differences in volume irradiated above 10 Gy between the plans were mostly small.

Fig. S5 (Supplementary Material) shows that $\mathrm{Vo}l_{\mathrm{DVH}}\left(0Gy\right)$ and $FI_{\left(0Gy,40Gy/s\right)}$ displayed the same trends as observed in Fig. 6 for $\mathrm{Vo}l_{\mathrm{DVH}}\left(10Gy\right)$ and $FI_{\left(10Gy,40Gy/s\right)}$. Furthermore, FLASH indices $FI_{\left(10Gy,100Gy/s\right)}$ and $FI_{\left(0Gy,100Gy/s\right)}$ showed in Figs. S6 and S7 (Supplementary Material), evidenced that the VHEE had lower $FI_{\left(10Gy,100Gy/s\right)}$ than protons. Regarding $FI_{\left(0Gy,100Gy/s\right)}$, VHEE at both energies displayed lower values than protons, except for the brain case for which the FI value was higher. Fig. S8 (Supplementary Material) showed for the brainstem and liver that there was no particularly clear trend in FI with the dose threshold, yet it was clear that higher dose rate thresholds imply lower FI values in all cases. For the liver, the FI was almost independent of the dose threshold between 5 Gy and 20 Gy. This trend was confirmed in Fig. S9 that displays the exhaustive FI variation with dose rate for all patient cases at fixed dose threshold of 10 Gy. In the explored dose and dose rate ranges (0–20 Gy, and 30–100 Gy/s), the dependence on the dose rate threshold was significantly stronger than on the dose threshold (Fig. S9, S10). This also generalizes well to OARs where the VolDVH was small (Fig. S11).

## 4 Discussion

In this work, equivalent transmission proton and VHEE plans were designed and compared. A newly defined FI helped in assessing protons UHDR coverage capacity to be higher than the VHEE observed one.

Slight differences found between the two VHEE energies suggest that VHEE’s are capable of achieving same performance as protons for the investigated treatment cases, in agreement with other studies [25], even if no specifically energy optimization was made [28]. Slightly lower mean doses at the OARs were often associated to the proton plans, maybe due to their sharper associated penumbra. Typically, better $D_{2\%}$ values were obtained with the VHEE 150 MeV plans and may be related to their depth dose profile that would offer higher dose to the target while better sparing the OAR compared to the flatter protons and VHEE 200 MeV depth dose.

Regarding dose calculation, potential source of numerical inaccuracy were identified to rely first in the use of a precomputed spot grid for the spot position optimization. Although an analytical model was employed for computing VHEE dose distributions, it has been shown to match Monte Carlo simulations with excellent accuracy and is therefore not expected to introduce any significant bias [30].

A limitation of this study is that the assumed parameters of the VHEE machine are based only on the preliminary published design of a future clinical accelerator [22]. In turn, this work provides important information regarding the machine parameters needed to attain a FLASH-compatible VHEE machine. If the beam current and maximum dose per pulse allow for the dose of each spot to be delivered within one pulse, then the PRF is the critical parameter that determines the irradiation time. A PRF of the order of 500 Hz is necessary to attain FLASH-compatible dose rates (≥40 Gy/s) at OARs for conventional fractionated treatments. An even higher PRF would be needed to equate the capabilities of the proton ProBeam system in terms of deliverable dose rates. Alternatively, wider spot-sizes could potentially allow for larger spacing between spots without loss of target coverage and homogeneity [51].

It should be noted, however, that the enormous difference in instantaneous dose rate between the proton and VHEE machines (assuming a cyclotron and a linear accelerator respectively) does not play a role under the adopted PBS dose rate definition [47]. Dose rate under non-continuous irradiation is ill-defined, and multiple other definitions can be found in literature [[52], [53], [54], [55]]. Therefore, results may vary under other PBS dose rate definitions. Nevertheless, recent indirect evidence suggests that the adopted PBS dose rate definition might correlate with biological effect [56].

The FLASH indices were in both cases relatively high, although the values for the proton plans were systematically higher than for the VHEE plans. The developed FLASH index relies on preset dose and dose rate thresholds, selected based on values reported in the literature. These values and parameters are very preliminary and only partially correlated with the incidence of the FLASH biological effect. We however explored alternative threshold values for assessing the FI robustness and demonstrated that the FLASH index depends significantly more on the dose rate than on the dose threshold, being almost independent of the dose threshold for OARs that significantly overlap the PTV. If the FI is intended to be used as a metric for FLASH-based planning, organ-specific dose and dose-rate thresholds should be established to account for differential tissue responses to the FLASH effect. As the body of research on FLASH radiotherapy continues to grow, we anticipate that a more robust consensus on these thresholds will emerge. Nevertheless, a FLASH index of one does not necessarily imply a strong FLASH effect: the OAR for which the FI equals one merely meets the UHDR conditions required for FLASH to occur, and only to a certain extent. These comparisons hold true if we consider that the radiobiological effect of FLASH does not depend on the radiation type. Although some studies suggest that the FLASH effect is similar between protons and electrons at low energies [25,57,58], no FLASH VHEE data is available. Furthermore, the FI dependence on dose and dose rate threshold means that it assumes at least partially cumulative effect over fields and fractions. It is not yet exactly understood how the time between fields and fractions affects FLASH effect and more experimental data is needed. The FLASH index defined in this work could serve as a quantitative metric for individual organs, thereby facilitating the optimization of FLASH-based radiotherapy treatment plans.

In summary, this study proposed a novel plan metric, the FLASH index, which does not depend on the magnitude of the radiobiological effect itself, offering a model-independent, quantitative metric to assess and compare the potential for FLASH delivery across treatment modalities, based solely on dose and dose rate criteria. Furthermore, our study identifies the pulse repetition frequency as a critical parameter for VHEE accelerators to achieve UHDRs comparable to those currently realized with proton therapy systems. Future work is warranted to investigate the impact of different beam structures and configurations on the dose rate and on the potential of VHEE in the context of FLASH.

## CRediT authorship contribution statement

**Flavia Gesualdi:** Data curation, Methodology, Writing – original draft, Software. **Louis Ermeneux:** Writing – review & editing, Software. **Pierre Lansonneur:** Methodology, Writing – review & editing, Software. **Mateusz Sitarz:** Writing – review & editing, Software. **Pierre Loap:** Writing – review & editing. **Gilles Créhange:** Writing – review & editing. **Anthony Magliari:** Writing – review & editing, Software. **Ludovic De Marzi:** Conceptualization, Methodology, Writing – review & editing, Supervision.

## Declaration of competing interest

The authors declare the following financial interests/personal relationships which may be considered as potential competing interests: P. Lansonneur and A. Magliari are employees at Varian, a Siemens Healthineers Company. L. De Marzi acknowledges support from a research grant by Varian.

## Footnotes

2 The referenced PBS dose rate definition necessitates a dose threshold, which was set to 0.01 Gy.
