# Supplementary material for: Comparison of protons and very high-energy electrons transmission pencil-beam-scanning for FLASH radiotherapy
Source: Phys Imaging Radiat Oncol. 2025 Nov 1;36:100860. doi: 10.1016/j.phro.2025.100860 (PMC12639469; doi:10.1016/j.phro.2025.100860)
Supplement: Supplementary Data 1 [file mmc1.docx]

Supplementary Material

Material and methods:

For the ProBeam system, the beam intensity is driven by the minimum Monitor Units (MU) in the energy layer, which was set to 600 MU/spot. This implied a beam current at the nozzle of approximately 215 nA, at which the corresponding MU rate was of 262.3x10^3^ MU/s. The speed of the scanning magnets was v_x_ = 5 mm/ms and v_y_ = 20 mm/ms. In the used configuration, if spots were more than 10 mm apart, the beam was interrupted between spots, otherwise it was rastered. For the VHEE system, the simulated beam current was set to 200 mA, and the maximum pulse duration was set to 3 µs.

Table S1: Plan characteristics of the selected patient cases. All beams were coplanar (couch angles of 0°).

| Case | PTV volume [cm^3^] | Prescribed dose [Gy] | Fractions | Gantry angles [°] | Number of spots |
| --- | --- | --- | --- | --- | --- |
| Brain metastasis | 4.3 | 18 | 1 | 313, 246, 205, 148, 90 | 29 |
| Lung | 13.8 | 45 | 3 | 306, 342, 18, 54 | 91 |
| Liver | 67.1 | 50 | 5 | 270, 232, 193, 158 | 126 |
| Prostate | 62.6 | 40 | 5 | 135, 225, 0 | 106 |

Tables S2 to S5 display the optimization objectives for the brain metastasis (1), lung (2), liver (3) and prostate (4) cases (abbreviations: “R.” for right, “L.” for left). The higher the maximum score of an objective, the higher its relative importance. The same optimization objectives were used for all treatment plans of a patient.

Table S2: Optimization objectives for the brain metastasis case.

| Structure | Metric | Maximum score criterion | Maximum score |
| --- | --- | --- | --- |
| PTV | Volume covered by 18.0 Gy | 99.5 % | 41 |
| PTV | Dose covering 99.5 % | 18.0 Gy | 25 |
| PTV | Dose covering 0.03 cm³ | 18.9 Gy | 18 |
| RingRx.1-1_PTV | Volume covered by 99.5 Gy | 0.0 cm³ | 5 |
| RingRx.1-1_PTV | Dose covering 0.03 cm³ | 17.91 Gy | 5 |
| RingMid.3-1.5_PTV | Dose covering 0.03 cm³ | 9.0 Gy | 8 |
| RingLow1.0_PTV | Volume covered by 6.0 Gy | 0.0 cm³ | 5 |
| RingLow1.0_PTV | Volume covered by 4.0 Gy | 0.0 cm³ | 5 |
| RingLow1.0_PTV | Volume covered by 2.0 Gy | 0.0 cm³ | 5 |
| Brainstem | Dose covering 1.0 cm³ | 1.0 Gy | 5 |
| Brainstem | Dose covering 0.03 cm³ | 2.0 Gy | 5 |
| Cochl L. | Dose covering 0.03 cm³ | 2.0 Gy | 5 |
| Cochl R. | Dose covering 0.03 cm³ | 2.0 Gy | 5 |
| Chiasm | Dose covering 0.2 cm³ | 2.0 Gy | 5 |
| Chiasm | Dose covering 0.03 cm³ | 2.0 Gy | 5 |
| OptNrv L. | Dose covering 0.2 cm³ | 2.0 Gy | 5 |
| OptNrv R. | Dose covering 0.2 cm³ | 2.0 Gy | 5 |
| OptNrv L. | Dose covering 0.03 cm³ | 2.0 Gy | 5 |
| OptNrv R. | Dose covering 0.03 cm³ | 2.0 Gy | 5 |
| Eye L. | Dose covering 0.03 cm³ | 0.5 Gy | 2 |
| Eye R. | Dose covering 0.03 cm³ | 0.5 Gy | 2 |
| Lens L. | Dose covering 0.03 cm³ | 0.5 Gy | 2 |
| Lens R. | Dose covering 0.03 cm³ | 0.5 Gy | 2 |

Table S3: Optimization objectives for the lung case.

| Structure | Metric | Maximum score criterion | Maximum score |
| --- | --- | --- | --- |
| PTV | Volume covered by 45 Gy | ≥ 95.5 % | 24 |
| PTV | Dose covering 99% | ≥ 45 Gy | 10 |
| PTV | Dose covering 0.035 cm³ | ≤ 48.38 Gy | 2.5 |
| NS Ring100 | Volume covered by 39.7 Gy | ≤ 0 % | 4 |
| NS Ring80 | Dose covering 0.035 cm³ | ≤ 18 Gy | 5 |
| External | Volume covered by 22.5 Gy | ≤ 40 cm³ | 5 |
| SpinalCord | Dose covering 0.035 cm³ | ≤ 4 Gy | 4 |
| SpinalCord | Dose covering 0.35 cm³ | ≤ 3.9 Gy | 4 |
| SpinalCord | Dose covering 1.2 cm³ | ≤ 1.7 Gy | 4 |
| Heart | Dose covering 15 cm³ | ≤ 5.25 Gy | 4 |
| Heart | Dose covering 0.035 cm³ | ≤ 7.9 Gy | 4 |
| Heart | Mean dose | ≤ 0 Gy | 1 |
| Skin | Dose covering 10 cm³ | ≤ 5.7 Gy | 4 |
| Skin | Dose covering 0.035 cm³ | ≤ 12.5 Gy | 4 |
| Lungs-GTV | Dose covering 1500 cm³ | ≤ 0 Gy | 4 |
| Lungs-GTV | Dose covering 1000 cm³ | ≤ 1.3 Gy | 4 |
| Lungs-GTV | Mean dose | ≤ 0 Gy | 1 |
| Lungs-GTV | Volume covered by 20 Gy | ≤ 2 % | 2 |
| Esophagus | Dose covering 0.035 cm³ | ≤ 3.6 Gy | 4 |
| Esophagus | Dose covering 5 cm³ | ≤ 4.5 Gy | 4 |
| Esophagus | Mean dose | ≤ 0 Gy | 1 |
| Tracheobronchial | Dose covering 0.035 cm³ | ≤ 18.75 Gy | 4 |
| Tracheobronchial | Dose covering 4 cm³ | ≤ 7.3 Gy | 4 |
| Tracheobronchial | Mean dose | ≤ 0 Gy | 1 |
| BrachialPlexus | Dose covering 0.035 cm³ | ≤ 11.8 Gy | 4 |
| BrachialPlexus | Dose covering 3 cm³ | ≤ 7.5 Gy | 4 |
| GreatVessels | Dose covering 0.035 cm³ | ≤ 21.2 Gy | 4 |
| GreatVessels | Dose covering 10 cm³ | ≤ 16.6 Gy | 4 |
| GreatVessels | Mean dose | ≤ 0 Gy | 1 |
| Stomach | Dose covering 0.035 cm³ | ≤ 10.4 Gy | 4 |
| Stomach | Dose covering 10 cm³ | ≤ 7.3 Gy | 4 |
| Stomach | Mean dose | ≤ 0 Gy | 1 |
| Ribs | Dose covering 0.035 cm³ | ≤ 17.2 Gy | 4 |
| Ribs | Dose covering 1 cm³ | ≤ 12.3 Gy | 4 |
| Ribs | Mean dose | ≤ 0 Gy | 1 |

Table S4: Optimization objectives for the liver case.

| Structure | Metric | Maximum score criterion | Maximum score |
| --- | --- | --- | --- |
| PTV | Dose covering 95.0 % | 50.0 Gy | 11 |
| PTV | Dose covering 99.0 % | 49.5 Gy | 11 |
| PTV | Dose covering 0.03 cm³ | 52.5 Gy | 11 |
| Liver-GTV | Mean dose | 7.5 Gy | 6 |
| Liver-GTV | Mean dose | 0.5 Gy | 5 |
| Liver-GTV | Dose covering 700.0 cm³ | 5.0 Gy | 10 |
| Esophagus | Dose covering 0.5 cm³ | 0.0 Gy | 6 |
| Stomach | Dose covering 0.5 cm³ | 10.0 Gy | 6 |
| Duodenum | Dose covering 0.5 cm³ | 10.0 Gy | 6 |
| Bowel | Dose covering 0.5 cm³ | 10.0 Gy | 6 |
| PTV Ring | Volume covered by 50.0 Gy | 0.0 % | 6 |
| PTV Ring | Dose covering 0.05 cm³ | 47.5 Gy | 6 |
| Spinal Cord PRV | Dose covering 0.5 cm³ | 10.0 Gy | 6 |
| Kidneys | Dose covering 33.0 % | 5.0 Gy | 6 |
| Kidneys | Mean dose | 5.0 Gy | 6 |
| Kidneys | Dose covering 10.0 % | 1.0 Gy | 6 |
| Heart | Dose covering 0.5 cm³ | 5.0 Gy | 6 |
| Ribs | Dose covering 2.0 % | 10.0 Gy | 6 |

Table S5: Optimization objectives for the prostate case.

| Structure | Metric | Maximum score criterion | Maximum score |
| --- | --- | --- | --- |
| PTV | Volume covered by 40.0 Gy | 100.0 % | 30 |
| PTV | Dose covering 99.99 % | 40.0 Gy | 12 |
| PTV | Dose covering 0.1 cm³ | 42.0 Gy | 8 |
| Rectum | Volume covered by 36.25 Gy | 0.1 cm³ | 4 |
| Rectum | Volume covered by 42.0 Gy | 0.03 cm³ | 3 |
| Rectum | Volume covered by 34.43 Gy | 1.0 cm³ | 3 |
| Rectum | Dose covering 50.0 % | 3.0 Gy | 10 |
| Rectum | Dose covering 20.0 % | 10.0 Gy | 6 |
| Rectum | Dose covering 1.0 cm³ | 34.0 Gy | 2 |
| Bladder | Volume covered by 36.25 Gy | 0.0 cm³ | 6 |
| Bladder | Volume covered by 42.0 Gy | 0.03 cm³ | 4 |
| Bladder | Volume covered by 32.62 Gy | 0.0 cm³ | 6 |
| Bladder | Dose covering 40.0 % | 3.0 Gy | 5 |
| Femoral Head L. | Volume covered by 19.9 Gy | 0.0 cm³ | 3 |
| Femoral Head R. | Volume covered by 19.9 Gy | 0.0 cm³ | 3 |
| PTV Ring | Dose covering 0.03 cm³ | 38.0 Gy | 5 |
| 50per. Ring | Volume covered by 20.0 Gy | 0.0 % | 7 |

Results:

For the lung case, the dose fall-off within the PTV was slightly better with protons, showing a 1 % advantage in CI_95_ compared to VHEE at both energies, and increased by 10 % and 16 % in HI_98_ relative to VHEE 200 MeV and 150 MeV, respectively. For the brain metastasis, the proton plan outperformed both VHEE energies in terms of conformity index, but VHEE scored higher in homogeneity by 10 % for 200 MeV and by 11 % for 150 MeV. The same trend was observed for the liver case, where the proton plan performs better in terms of conformity but worse in homogeneity compared to VHEE. Specifically, for VHEE 150 MeV, the HI_98_ was improved by 7 % while the CI_95_ was 8 % lower relative to protons; for VHEE 200 MeV, the HI_98_ was 5 % improved while the CI_95_ was 5% lower compared to protons. Finally, for the prostate case, VHEE outperformed protons in CI_95_ and HI_98_ for both energies, with the best CI_95_ achieved by VHEE 150 MeV (4% higher than for protons), and the best HI_98_ achieved by VHEE 200 MeV (5% higher than for protons). However, protons still achieved the best performance in CI_50_ in this case

For the brain case, the proton plan provided superior sparing of all OARs except for the right optic nerve, where VHEE 150 MeV yielded the lowest D_2_ and VHEE 200 MeV achieved the lowest D_mean_. In the lung case, only the spinal cord benefited from better sparing with VHEE 150 MeV, showing a 10% reduction in D_2_, while protons performed better for all other OARs. In the liver case, VHEE 150 MeV provided the lowest D_2_ and D_mean_ for the vena cava, stomach, and bowel. For the remaining OARs, the best values for these metrics was shared between protons and VHEE at both energies. Finally, in the prostate case, protons generally achieved the lowest D_2_ and D_mean_ across most OARs, except for the rectum, where both VHEE energies resulted in slightly lower D_2_ values by approximately 1%.

A thorough comparison between the VHEE 200 MeV and 150 MeV treatment plans is presented hereafter. Figure S1 shows the comparison of the isodose curves, Figure S2 compares the DVHs, and Figure S3 presents the comparison of the 10 Gy isodose contour against the 10 Gy isodose contour delivered at a dose rate ≥ 40 Gy/s (assuming a pulse repetition frequency of 500 Hz). These are equivalent to Figures 2, 3, and 5 of the main manuscript respectively, and can be easily compared taking the VHEE 200 MeV plan as a reference, since it is exactly the same between the corresponding figures.

For the brain and lung cases, the $FI_{(10 Gy, 40 Gy/s)}$ values were very close to one for all investigated modalities, but for the right cochlea in which the FI was lower for VHEE 150 MeV than VHEE 200 MeV, and was ill-defined for protons. For the liver and prostate cases, the $FI_{(10 Gy, 40 Gy/s)}$ values were below one, but close to for protons. VHEE as for the lung and brain cases were showing lower FI values than protons for both patient cases. $Vol_{DVH}(10Gy)$ varied between protons and VHEE, but stayed close for both VHEE energies.


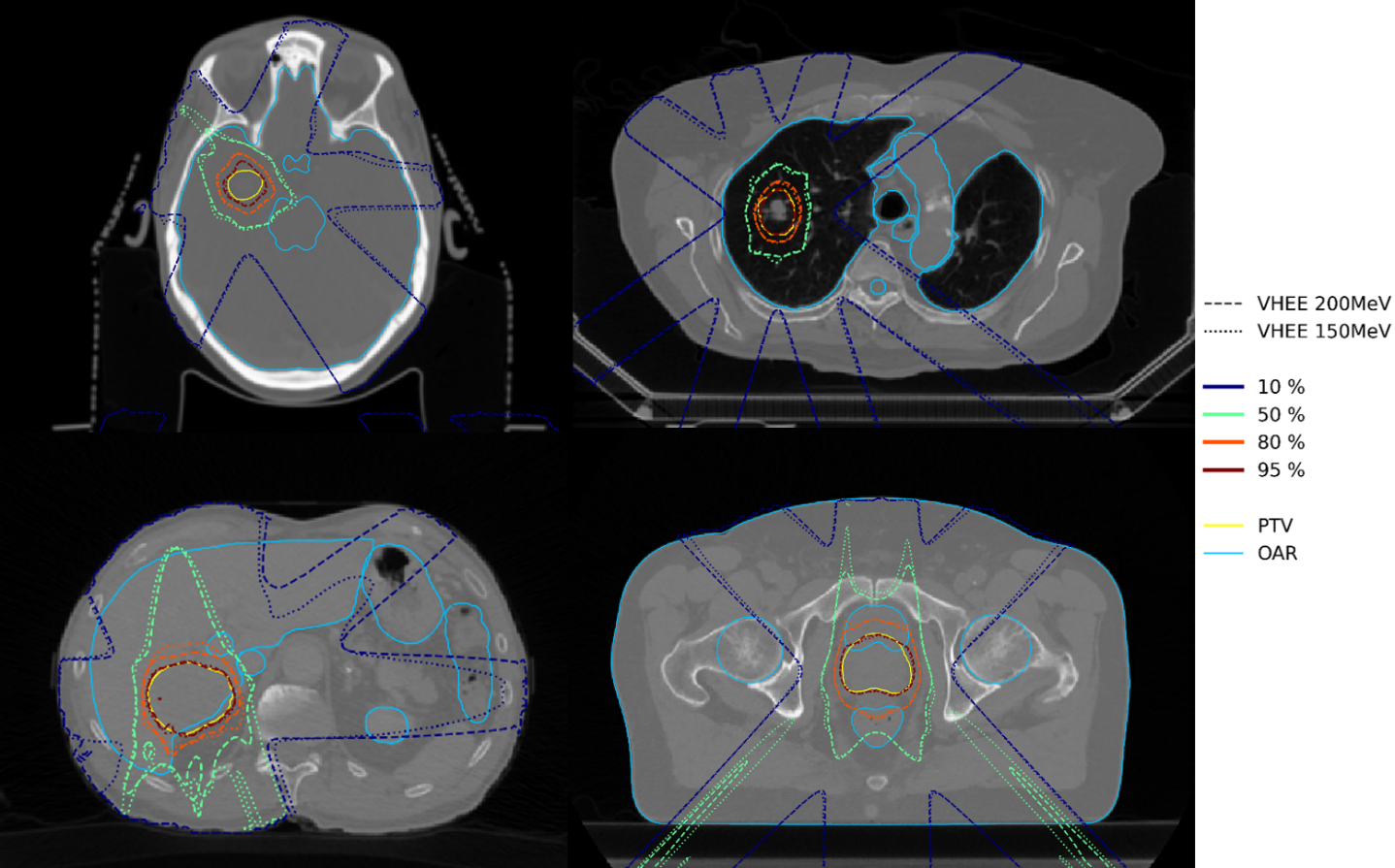


Figure S1: Isodose curves (relative to the prescribed dose, 10% in blue, 50% in green, 80% in orange, and 95% in red) for the brain metastasis (upper left), lung (upper right), liver (lower left), and prostate (lower right) cases. The VHEE 200 MeV plans are displayed in dashed lines and the VHEE 150 MeV in dotted lines.


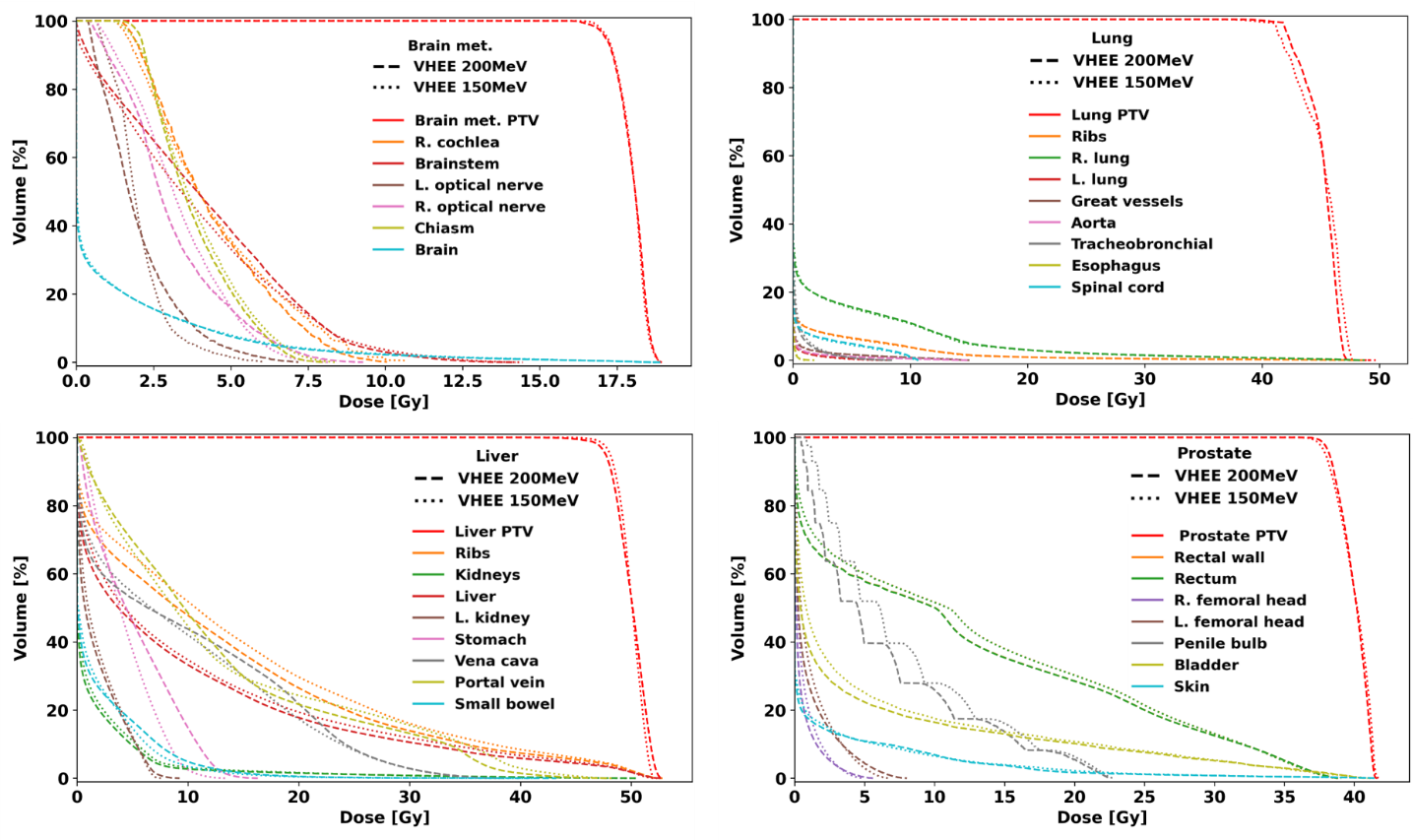


Figure S2: DVH comparison for the brain metastasis (upper left), lung (upper right), liver (lower left), and prostate (lower right) cases. The VHEE 200 MeV plans are displayed in dashed lines and the VHEE 150 MeV in dotted lines. Abbreviations: “R.” for right, “L.” for left.


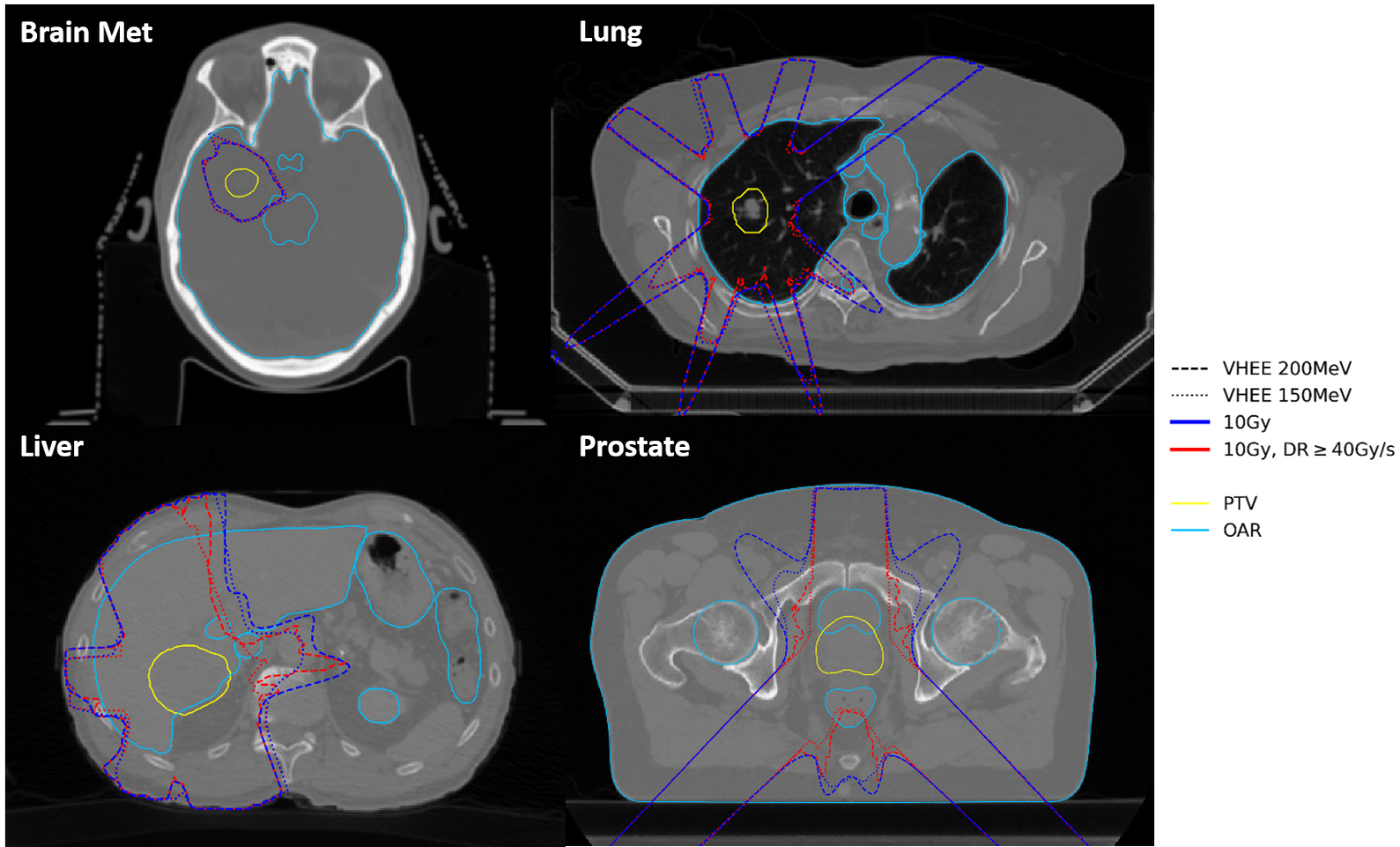


Figure S3: 10 Gy isodose contour (blue) against the 10 Gy isodose contour delivered at a dose rate ≥ 40 Gy/s for a PRF of 500 Hz (red), for each case, for VHEE at 200 MeV (dashed lines) and VHEE at 150 MeV (dotted lines).


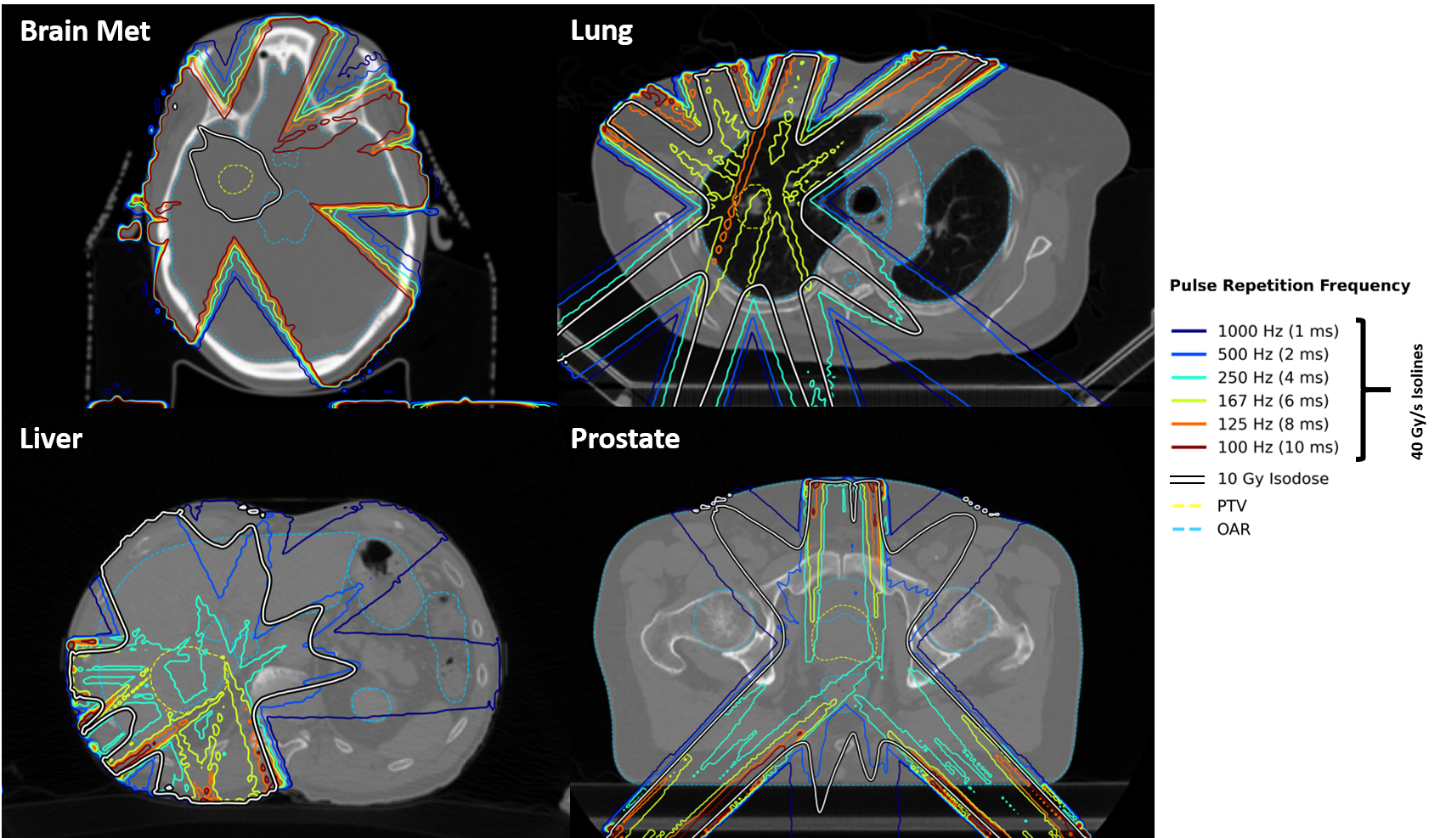


Figure S4: Comparison of the 10 Gy isodose curves (solid black lines), and the isolines for dose rate ≥ 40 Gy/s for more pulse repetition frequencies (solid color lines), considering VHEEs at 200 MeV, for the brain metastasis (upper left), lung (upper right), liver (lower left), and prostate (lower right) cases.

Moreover, a FLASH index without a dose threshold,$FI_{0}$, is defined for each structure of each treatment plan as the number of voxels receiving dose (≥ 0 Gy) at a dose rate ≥40 Gy/s, $N_{vox,D>0Gy,DR\geq40Gy/s}$, divided by the total number of voxels of the structure receiving dose ≥ 0 Gy:

$FI_{(0 Gy, 40 Gy/s)}=\frac{N_{vox, D>0Gy,DR\geq40Gy/s}}{N_{vox,D>0Gy}}=\frac{Vol_{DRDVH}\left( 0Gy, 40 Gy/s \right)}{Vol_{DVH}\left( 0Gy \right)}$ (1)

Figure S5 shows the percentage of the volume of the organs at risk receiving dose (>0 Gy), $Vol_{DVH}(0Gy)$, and the FLASH index $FI_{(0 Gy, 40 Gy/s)}$, for all considered organs at risk, for the four patients, for the three treatment plans. This is equivalent to Fig. 5 of the main article, but without considering a dose threshold.


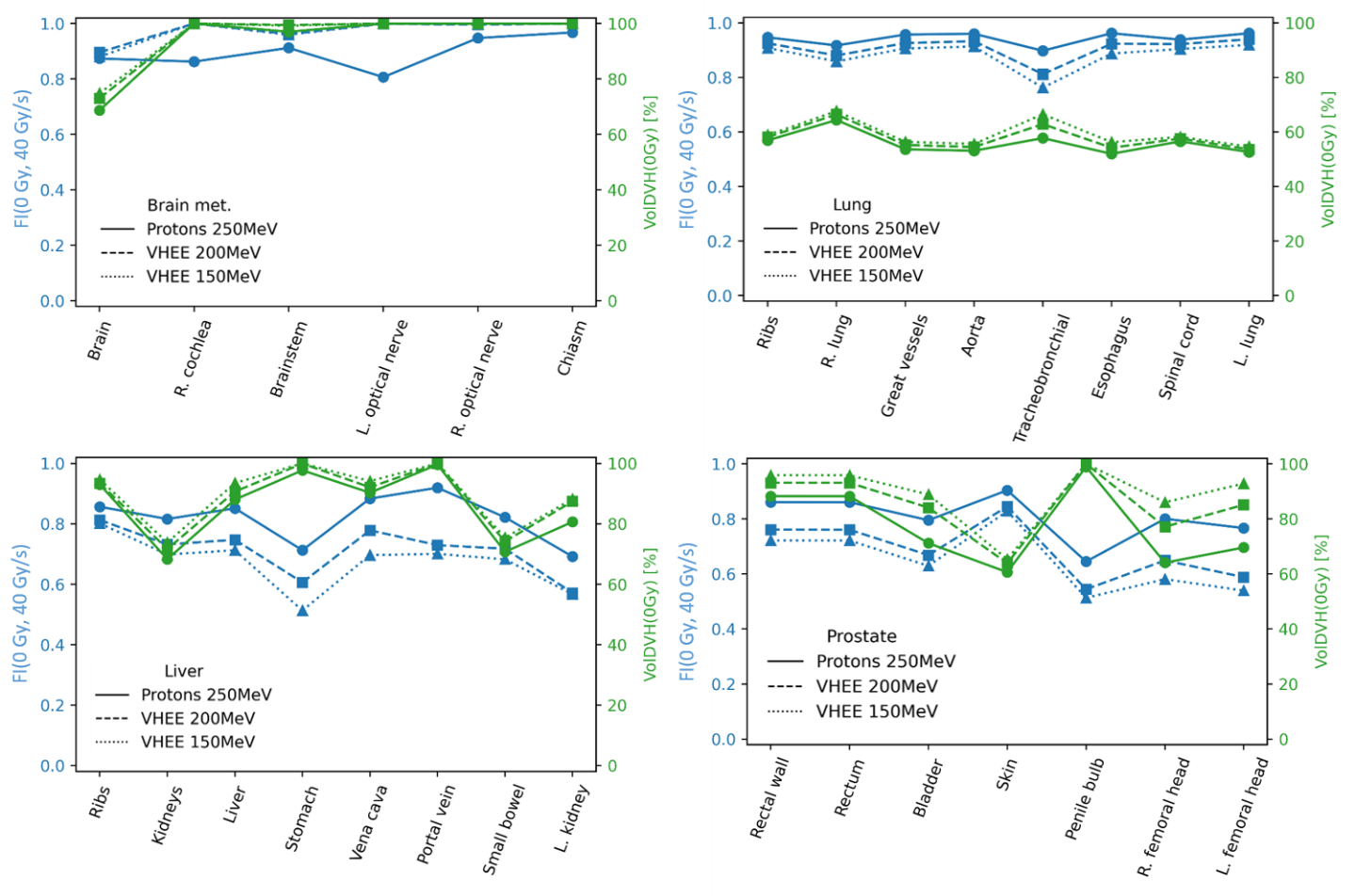


Figure S5: Comparison of the FLASH index $FI_{(0 Gy, 40 Gy/s)}$(c.f. Eq. (1), left y-axis, blue) at a dose rate threshold of 40 Gy/s and of $Vol_{DVH}(0Gy)$ (green, right y-axis), for the brain metastasis (upper left), lung (upper right), prostate (lower left), and liver (lower right) cases. Proton 250 MeV plans are shown in solid lines, the VHEE 200 MeV in dashed lines, and the VHEE 150 MeV in dotted lines.

The FI index could be used to differentiate plans between those fulfilling UHDR conditions for FLASH, and those, which do not. In the case in which the percentage of the volume receiving 10 Gy or more ($Vol_{DVH}(10Gy))$ is equal or smaller in the plan with higher FLASH index, then the latter plan is overall better for this OAR. If the plan with a higher FLASH index also has a higher $Vol_{DVH}(10Gy)$ for the OAR in question, then the relative radiobiological effect of FLASH should be known to estimate which plan is overall better. Equivalent FLASH indices to $FI_{(10 Gy, 100 Gy/s)}$and $FI_{(0 Gy, 100 Gy/s)}$ can be defined using as dose threshold 100 Gy/s instead of 40 Gy/s. These are shown in Figs. S6 and S7 respectively. It is noteworthy that the values of $Vol_{DVH}(0Gy)$ and $Vol_{DVH}(10Gy)$, shown in the right y-axes, are independent of the selected dose rate threshold.


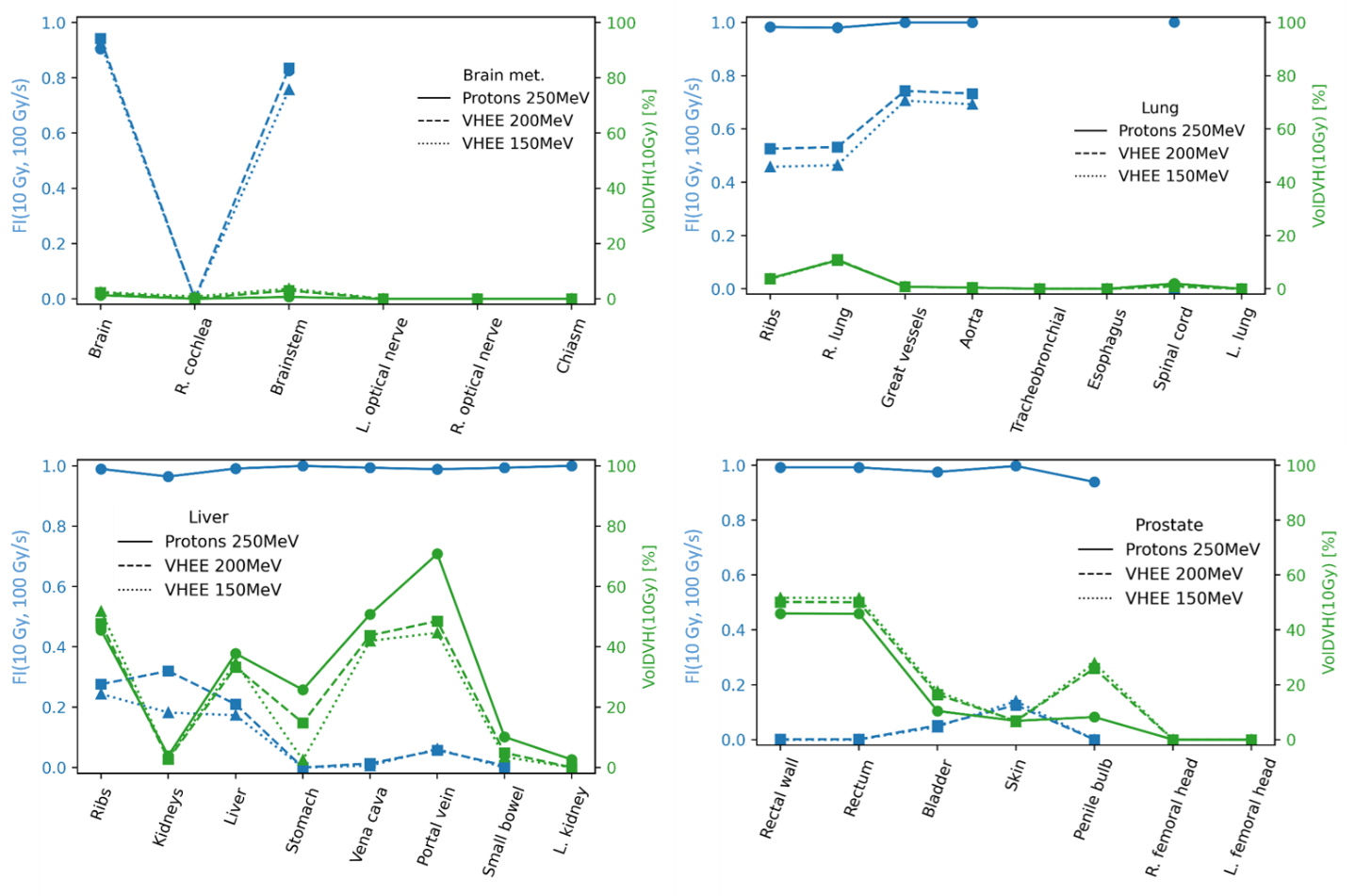


Figure S6: Comparison of the FLASH index $FI_{(10 Gy, 100 Gy/s)}$ (c.f. Eq. (1), blue, left y-axis) at a dose rate threshold of 100 Gy/s, and volume irradiated above 10 Gy, $Vol_{DVH}(10Gy)$ (green, right y-axis), for the brain metastasis (upper left), lung (upper right), liver (lower left), and prostate (lower right) cases. Proton 250 MeV plans are shown in solid lines, the VHEE 200 MeV in dashed lines, and the VHEE 150 MeV in dotted lines.


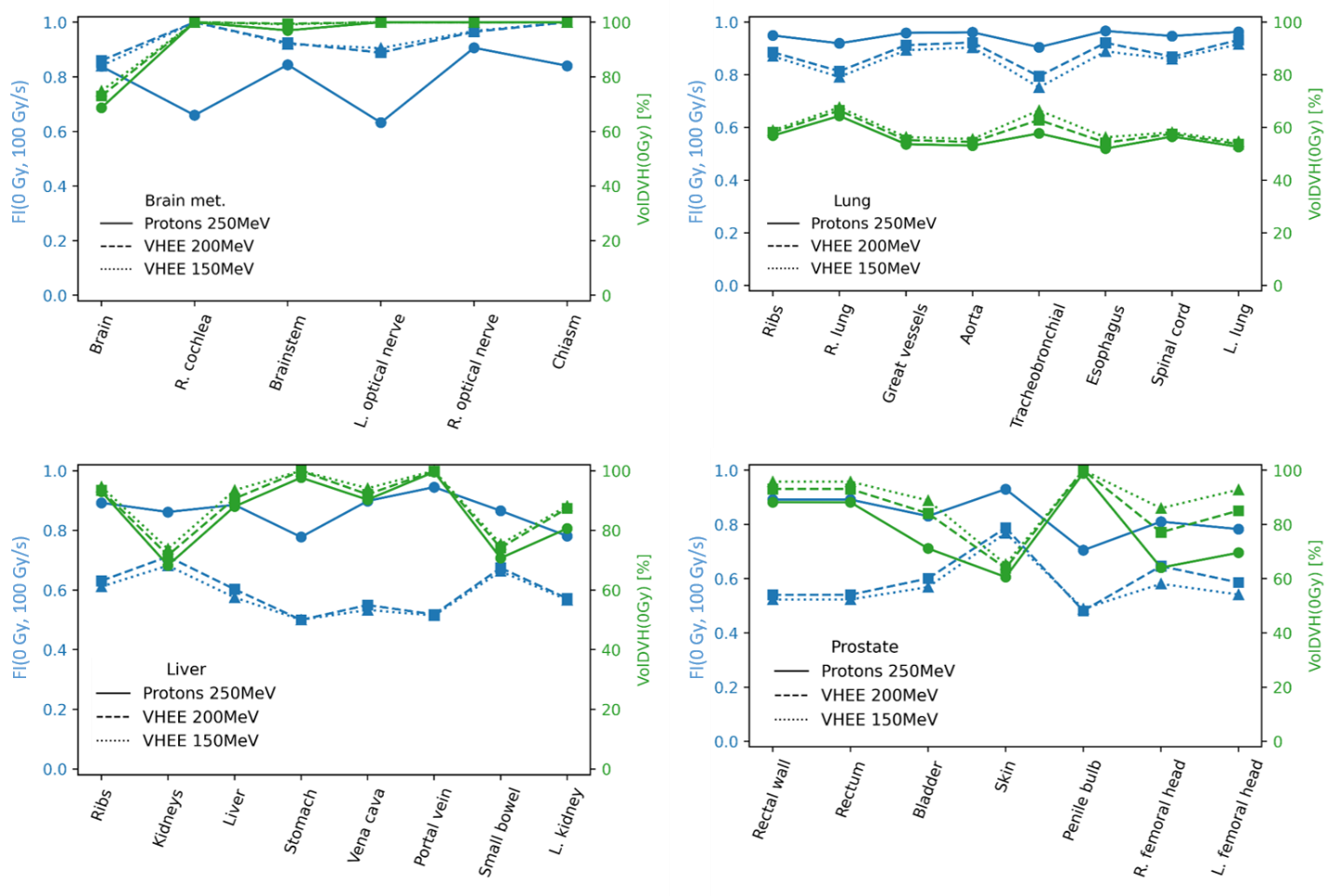


Figure S7: Comparison of the FLASH index $FI_{(0 Gy, 100 Gy/s)}$ (c.f. Eq. (1), left y-axis, blue) at a dose rate threshold of 100 Gy/s and of $Vol_{DVH}(0Gy)$ (green, right y-axis), for the brain metastasis (upper left), lung (upper right), prostate (lower left), and liver (lower right) cases. Proton 250 MeV plans are shown in solid lines, the VHEE 200 MeV in dashed lines, and the VHEE 150 MeV in dotted lines.

An analysis of the influence of the dose and dose rate thresholds in the FLASH index is shown in Fig. S8. To illustrate the general trends, two example volumes were chosen: the brainstem of the brain case, because it is very close to the dose fall-off but outside of the PTV, and the liver of the liver case, because the PTV falls almost entirely inside of it.


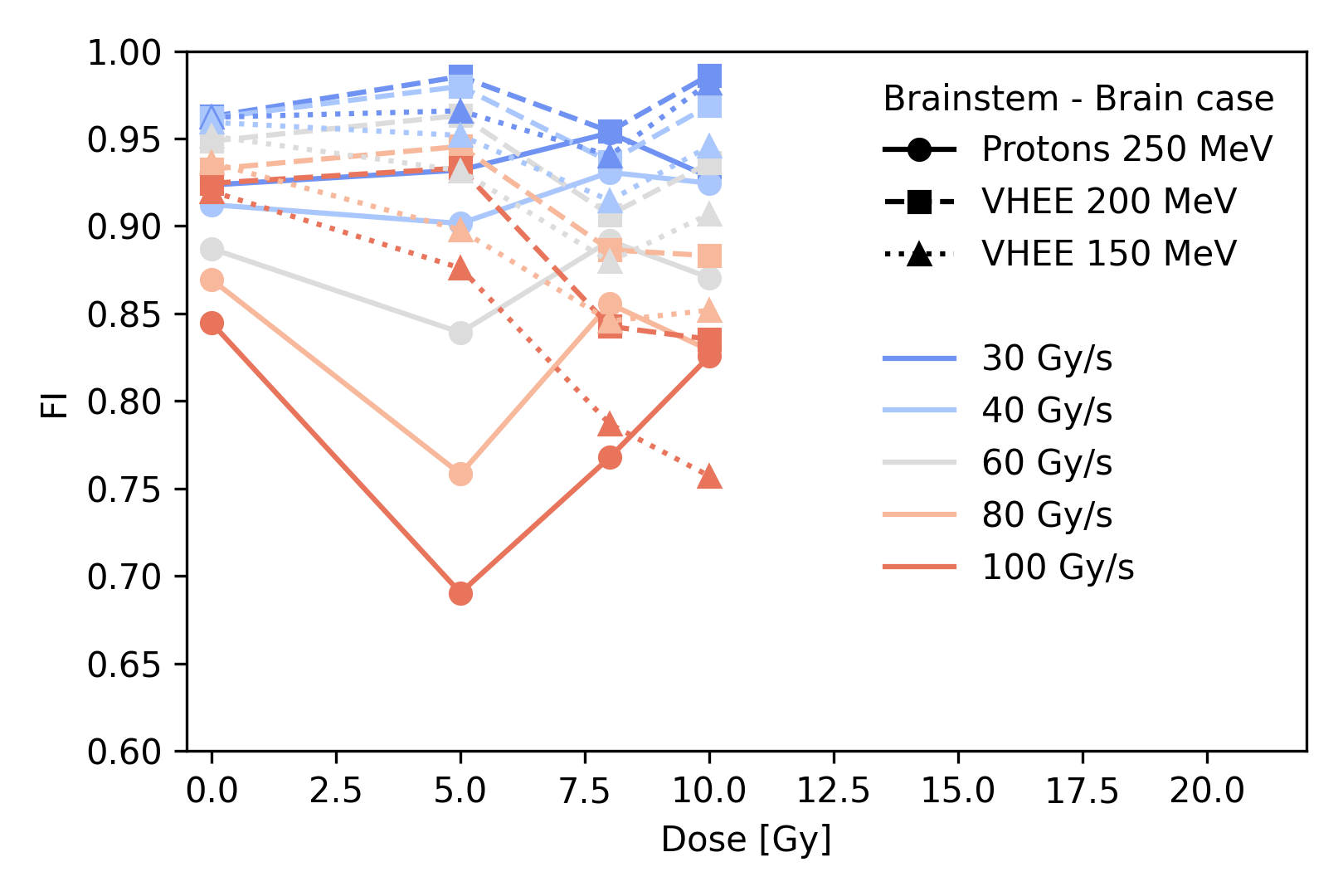

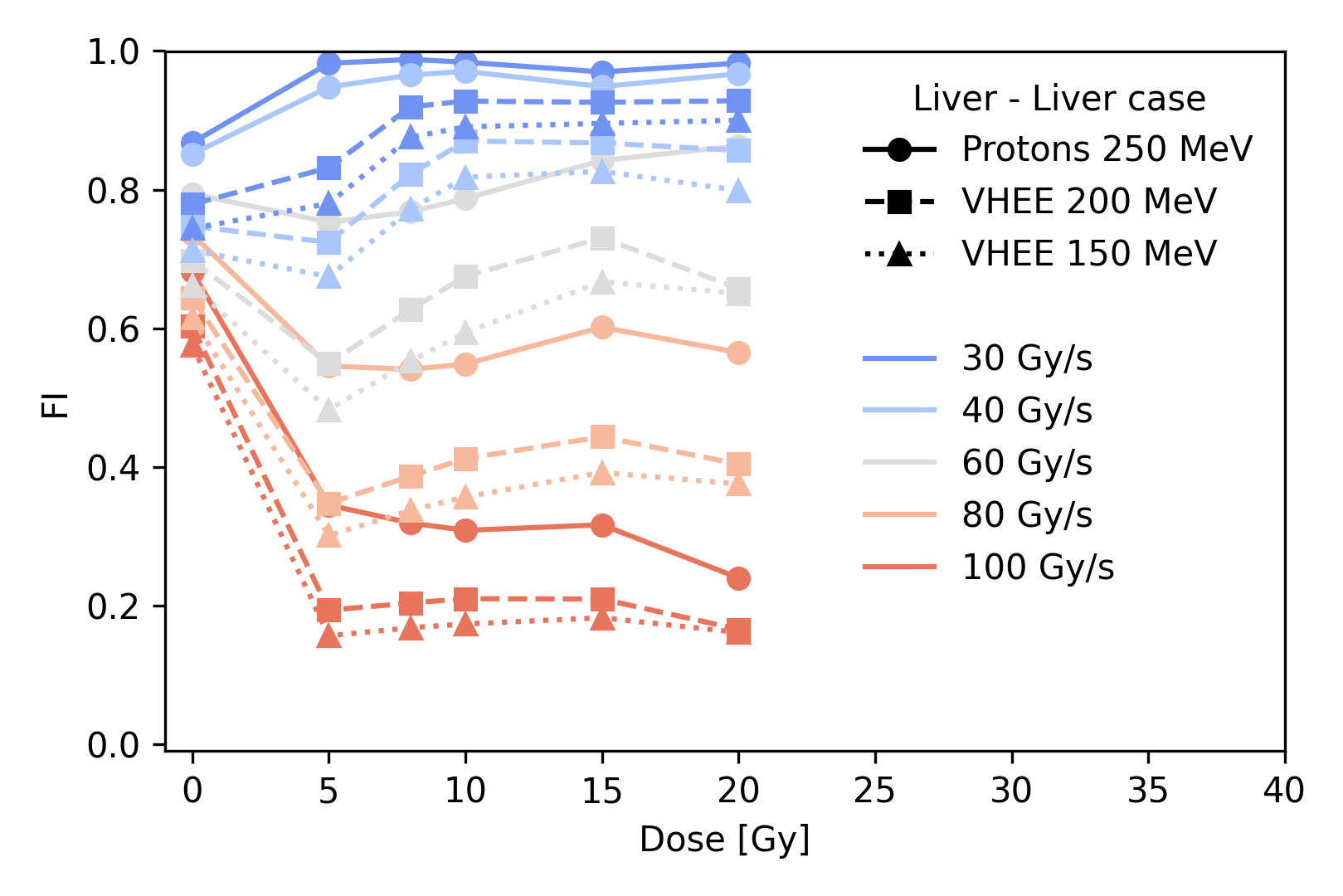

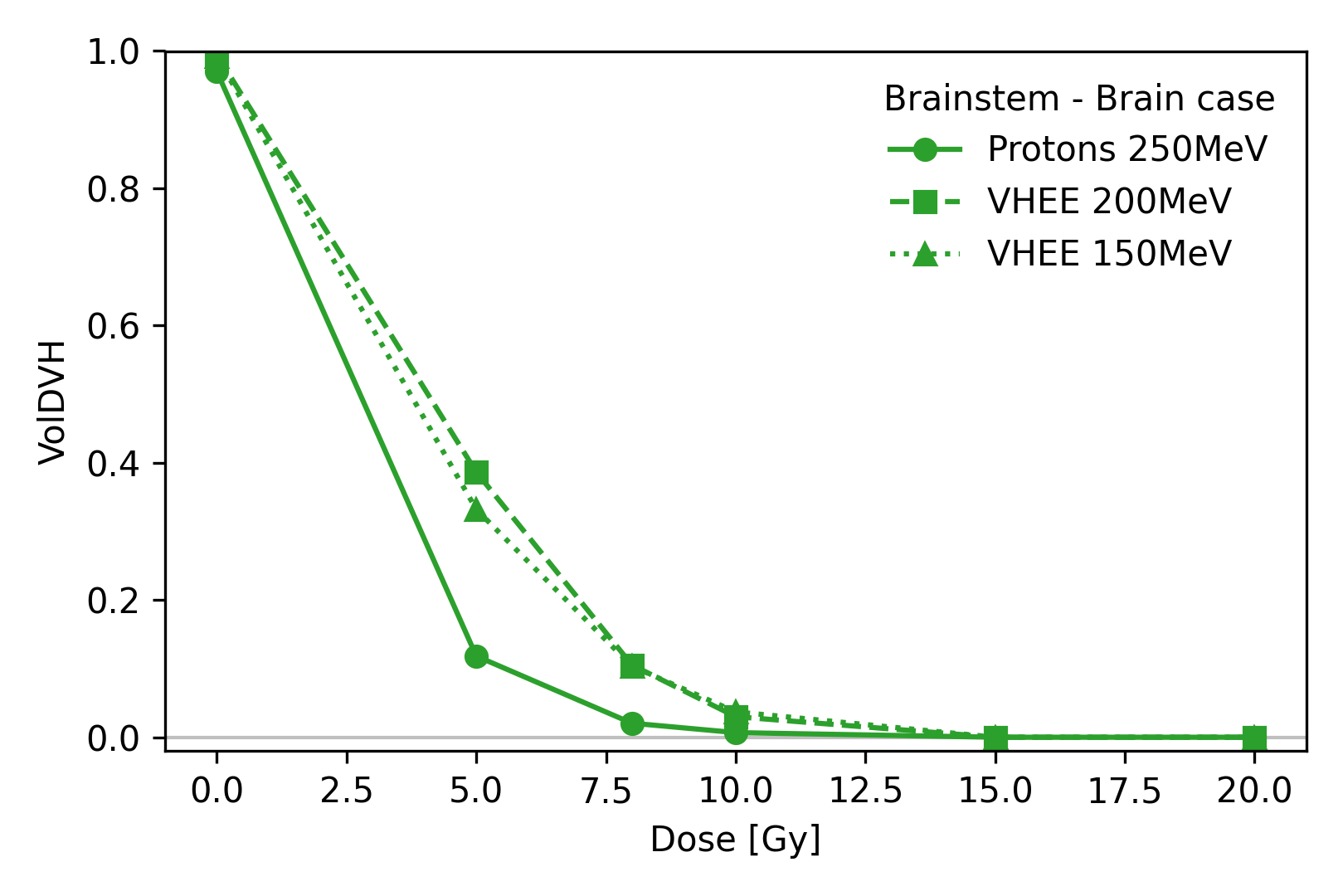

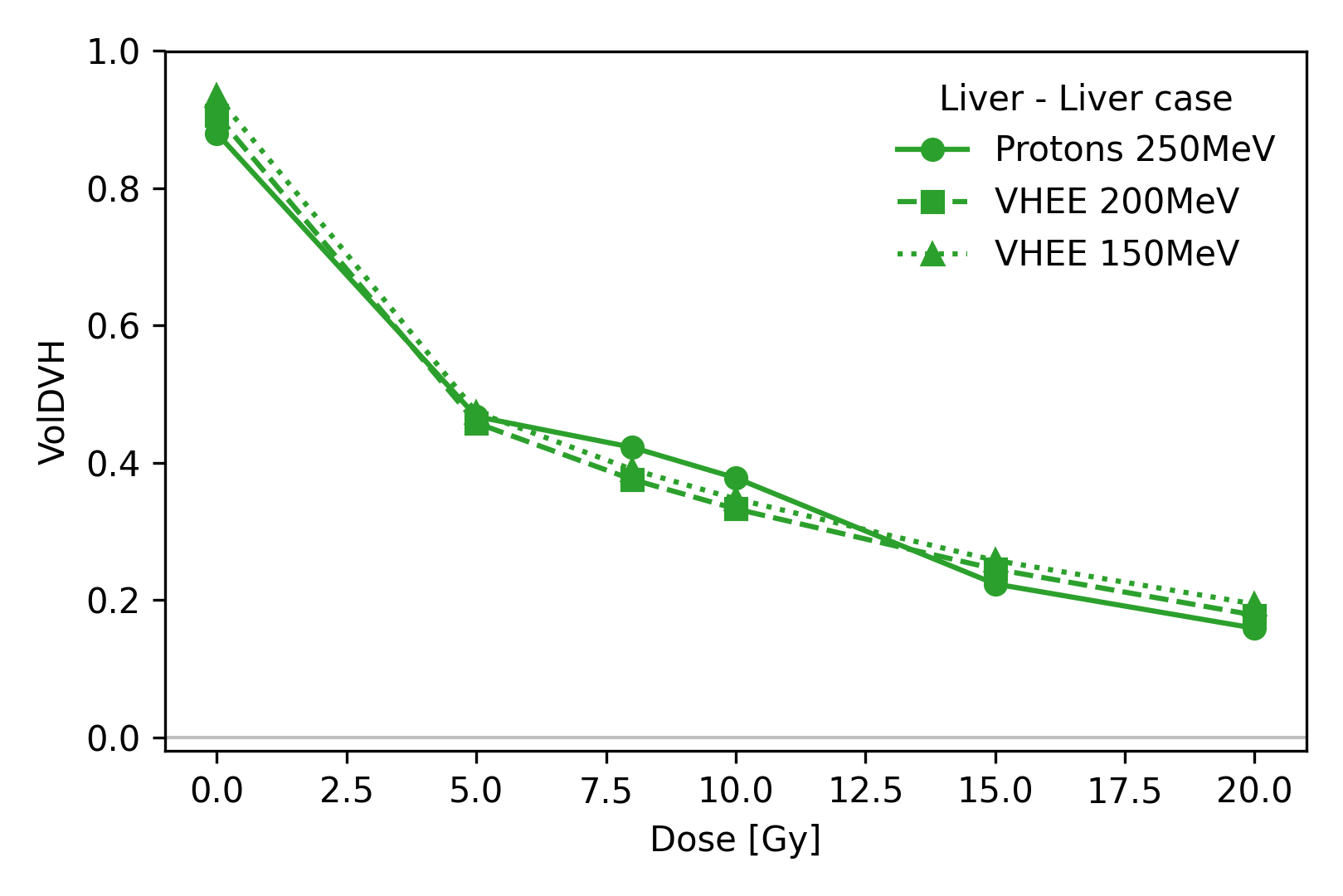


Figure S8: The two top figures show the FLASH index as a function of the dose threshold (x-axis) for different dose-rate thresholds (different line colors), for protons at 250 MeV (solid lines), VHEE 200 MeV (dashed lines) and VHEE 150 MeV (dotted lines). The two bottom figures show the percentage of the volume receiving more than the dose in the x-axis. Again, different line types correspond to the different irradiation modalities. The left figures correspond to the brainstem of the brain case, while the right figures to the liver of the liver case.

Figure S9, S10 and S11 present for all patients cases the behavior of the $FI_{(10 Gy, X Gy/s)}$ for a fixed dose of 10 Gy and varying dose-rate (Figure S9), $FI_{(X Gy, 40 Gy/s)}$ for a fixed dose-rate of 40 Gy/s and varying doses (Figure S10), and of the $Vol_{DVH}\left( X Gy \right)$ for several dose thresholds (Figure S11).


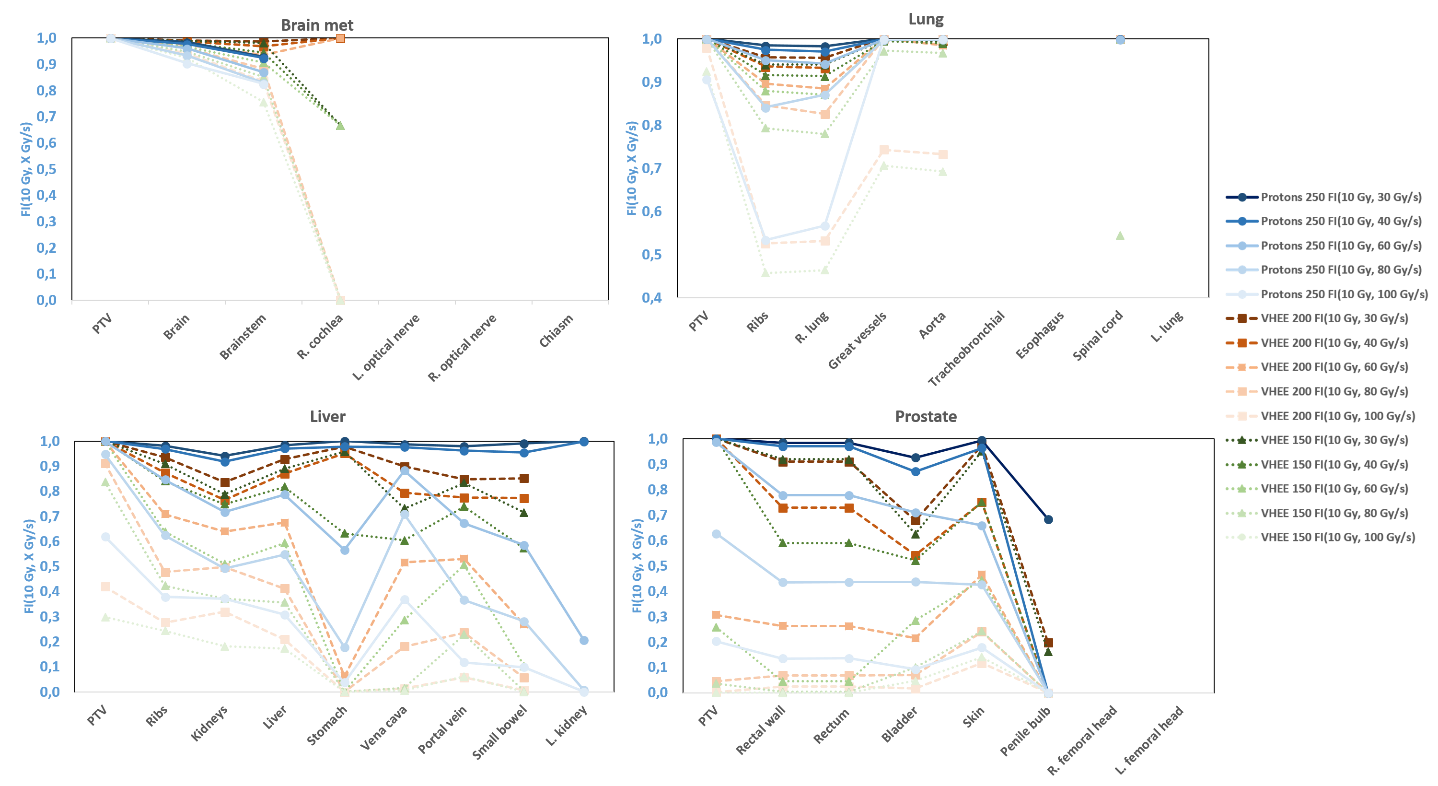


Figure S9 : $FI_{(10 Gy, X Gy/s)}$ values for a fixed dose of 10 Gy and varying dose-rate for all patient cases and all related structure.


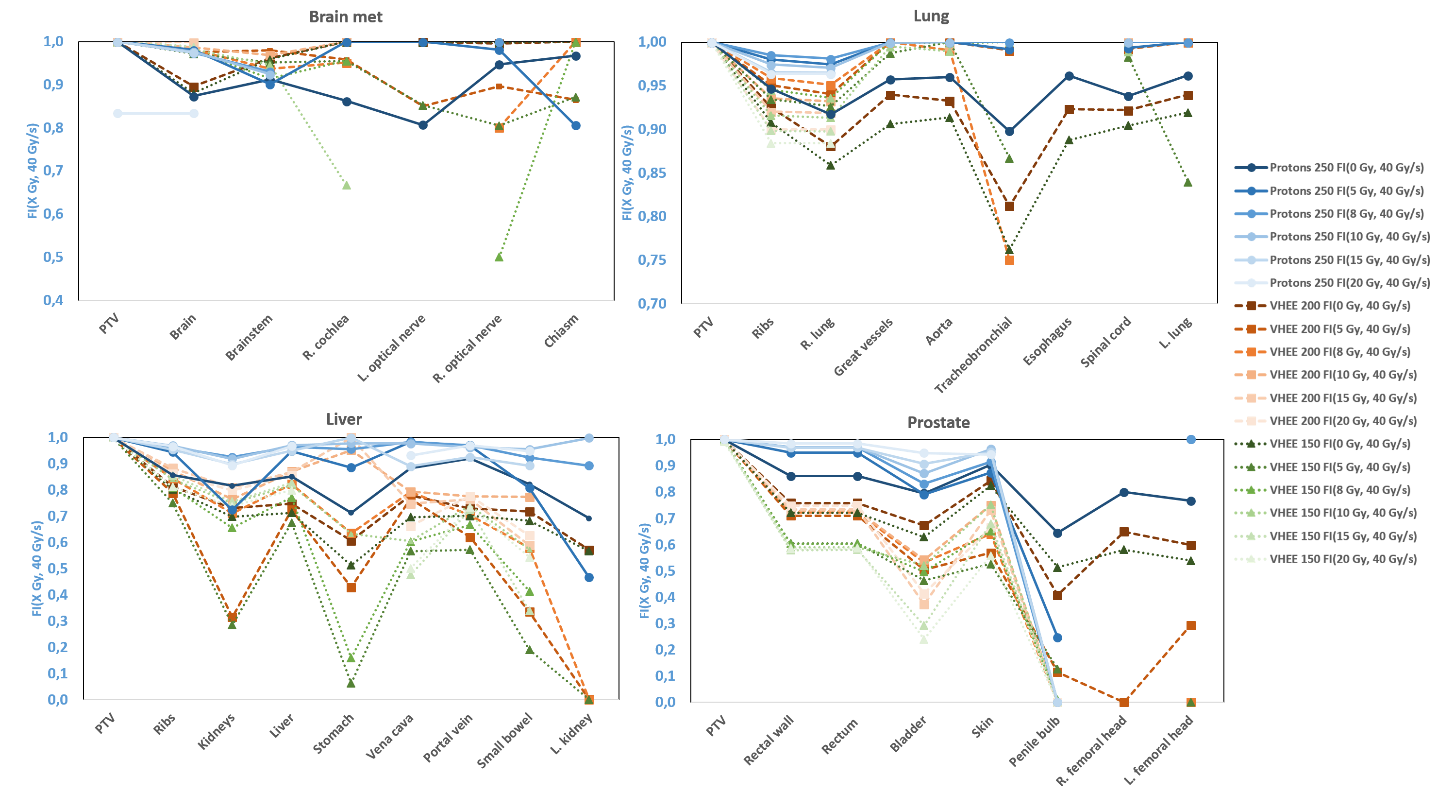


Figure S10 : $FI_{(X Gy, 40 Gy/s)}$ values for a fixed dose-rate of 40 Gy/s and varying doses for all patient cases and all related structure.


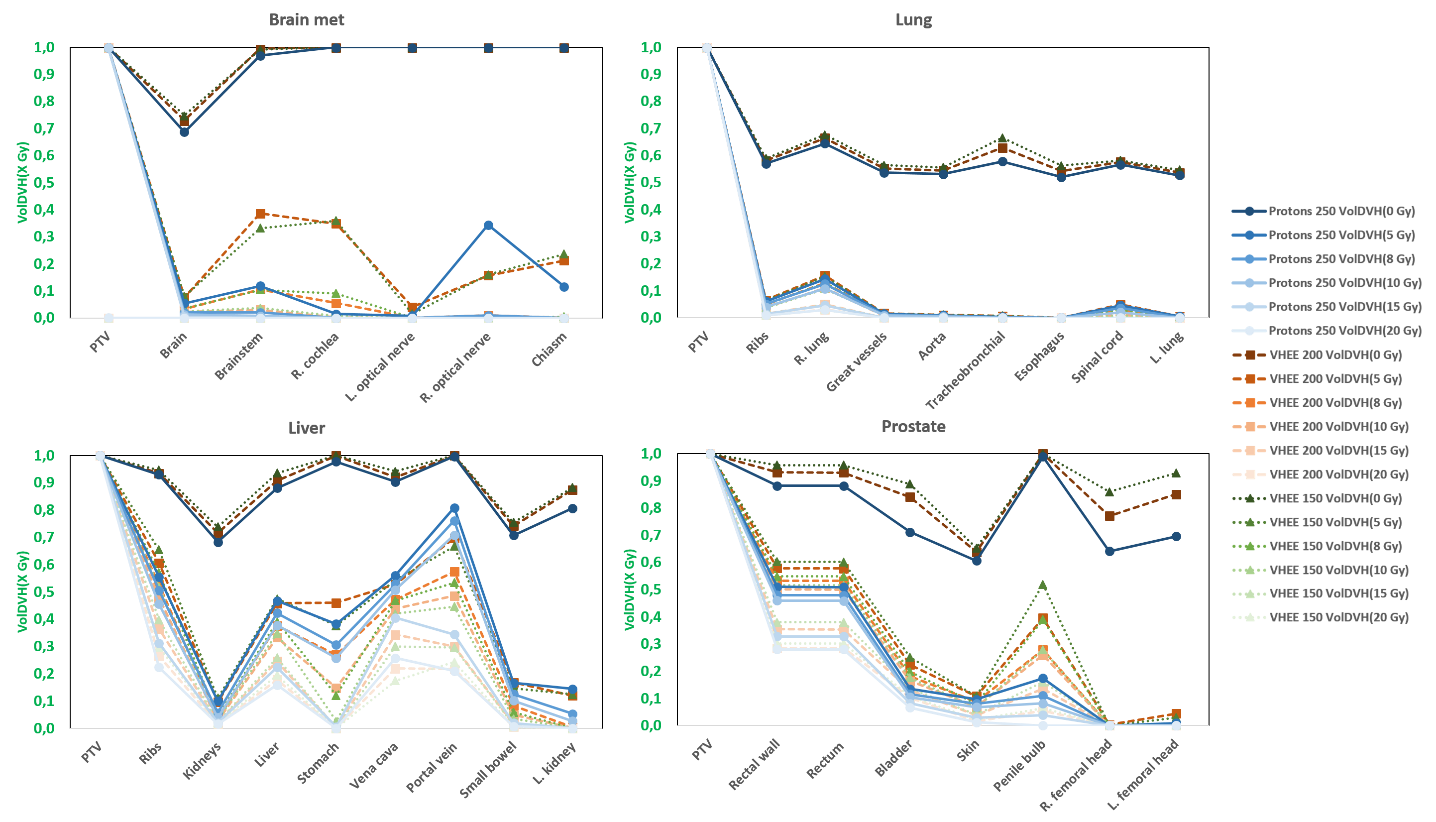


Figure S11 : $Vol_{DVH}\left( X Gy \right)$ values for several dose thresholds for all patient cases and all related structure.

Furthermore, Tables S6 to S9 present the plan metrics for the brain metastasis (5), lung (6), liver (7) and prostate (8) cases. For the PTVs, the conformity indices CI_50_ and CI_95_ are reported, as well as the homogeneity index HI_98_, and the dose quantiles $D_{2\%}$, $D_{95\%}$, and $D_{98\%}$. For the organs at risk, the $D_{2\%}$ and $D_{\mathrm{mean}}$ are reported.

Table S6: Plan metrics for the brain metastasis case.

|  | Brain met. PTV | | | | | |
| --- | --- | --- | --- | --- | --- | --- |
|  | CI_50_ | CI_95_ | HI_98_ | D_2%_ | D_95%_ | D_98%_ |
| Protons 250MeV | 0.191 | 0.946 | 0.816 | 19.560 | 16.321 | 15.961 |
| VHEE 200MeV | 0.112 (-41 %) | 0.721 (-24 %) | 0.899 (+10 %) | 18.746 (-4 %) | 17.128 (+5 %) | 16.847 (+6 %) |
| VHEE 150MeV | 0.099 (-48 %) | 0.714 (-25 %) | 0.905 (+11 %) | 18.721 (-4 %) | 17.191 (+5 %) | 16.941 (+6 %) |

|  | Brain | | Brainstem | | Chiasm | |
| --- | --- | --- | --- | --- | --- | --- |
|  | D_2%_ | D_mean_ | D_2%_ | D_mean_ | D_2%_ | D_mean_ |
| Protons 250MeV | 7.954 | 0.768 | 8.055 | 2.346 | 6.131 | 2.799 |
| VHEE 200MeV | 10.555 (+33 %) | 1.158 (+51 %) | 10.693 (+33 %) | 4.147 (+77 %) | 6.933 (+13 %) | 3.783 (+35 %) |
| VHEE 150MeV | 11.023 (+39 %) | 1.188 (+55 %) | 11.146 (+38 %) | 3.893 (+66 %) | 7.280 (+19 %) | 3.877 (+39 %) |

|  | R. cochlea | | L. optical nerve | | R. optical nerve | |
| --- | --- | --- | --- | --- | --- | --- |
|  | D_2%_ | D_mean_ | D_2%_ | D_mean_ | D_2%_ | D_mean_ |
| Protons 250MeV | 4.808 | 1.360 | 4.438 | 1.187 | 7.466 | 3.619 |
| VHEE 200MeV | 8.864 (+84 %) | 4.392 (+223 %) | 5.713 (+29 %) | 2.012 (+70 %) | 7.464 (0 %) | 3.092 (-15 %) |
| VHEE 150MeV | 9.388 (+95 %) | 4.460 (+228 %) | 4.804 (+8 %) | 2.029 (+71 %) | 6.725 (-10 %) | 3.307 (-9 %) |

Table S7: Plan metrics for the lung case.

|  | Lung PTV | | | | | | |
| --- | --- | --- | --- | --- | --- | --- | --- |
|  | CI_50_ | CI_95_ | | HI_98_ | D_2%_ | D_95%_ | D_98%_ |
| Protons 250MeV | 0.233 | 0.935 | | 0.887 | 47.385 | 42.563 | 42.037 |
| VHEE 200MeV | 0.219 (-6 %) | 0.923 (-1 %) | | 0.804 (-9 %) | 48.386 (+2 %) | 40.336 (-5 %) | 38.893 (-7 %) |
| VHEE 150MeV | 0.224 (-4 %) | 0.926 (-1 %) | | 0.765 (-14 %) | 48.247 (+2 %) | 38.148 (-10 %) | 36.919 (-12 %) |
|  |  |  | |  |  |  |  |
|  |  |  | |  |  |  |  |
|  | Aorta | | Esophagus | | | Great vessels | |
|  | D_2%_ | D_mean_ | D_2%_ | | D_mean_ | D_2%_ | D_mean_ |
| Protons 250MeV | 0.723 | 0.119 | 0.007 | | 0.002 | 2.170 | 0.174 |
| VHEE 200MeV | 1.634 (+126 %) | 0.146 (+23 %) | 0.208 (+3E3 %) | | 0.015 (+650 %) | 3.433 (+58 %) | 0.219 (+26 %) |
| VHEE 150MeV | 1.700 (+135 %) | 0.154 (+29 %) | 0.262 (+4E3 %) | | 0.020 (+900 %) | 3.642 (+68 %) | 0.230 (+32 %) |

|  | L. lung | | R. lung | | Ribs | |
| --- | --- | --- | --- | --- | --- | --- |
|  | D_2%_ | D_mean_ | D_2%_ | D_mean_ | D_2%_ | D_mean_ |
| Protons 250MeV | 0.318 | 0.067 | 24.401 | 2.367 | 12.646 | 0.897 |
| VHEE 200MeV | 1.136 (+257 %) | 0.081 (+21 %) | 25.194 (+3 %) | 2.563 (+8 %) | 13.595 (+8 %) | 0.975 (+9 %) |
| VHEE 150MeV | 1.156 (+264 %) | 0.080 (+19 %) | 24.682 (+1 %) | 2.520 (+6 %) | 13.335 (+5 %) | 0.956 (+7 %) |

|  | Spinal cord | | Tracheobronchial | |
| --- | --- | --- | --- | --- |
|  | D_2%_ | D_mean_ | D_2%_ | D_mean_ |
| Protons 250MeV | 9.981 | 0.487 | 1.289 | 0.087 |
| VHEE 200MeV | 9.334 (-6 %) | 0.514 (+6 %) | 2.972 (+131 %) | 0.211 (+143 %) |
| VHEE 150MeV | 8.940 (-10 %) | 0.489 (+0 %) | 3.217 (+150 %) | 0.253 (+191 %) |

Table S8: Plan metrics for the liver case.

|  | PTV Liver | | | | | |
| --- | --- | --- | --- | --- | --- | --- |
|  | CI_50_ | CI_95_ | HI_98_ | D_2%_ | D_95%_ | D_98%_ |
| Protons 250MeV | 0.247 | 0.957 | 0.859 | 53.521 | 46.693 | 45.949 |
| VHEE 200MeV | 0.194 (-21 %) | 0.911 (-5 %) | 0.898 (+5 %) | 52.284 (-2 %) | 47.697 (+2 %) | 46.956 (+2 %) |
| VHEE 150MeV | 0.144 (-42 %) | 0.877 (-8 %) | 0.917 (+7 %) | 51.784 (-3 %) | 48.103 (+3 %) | 47.490 (+3 %) |

|  | Kidneys | | Left kidney | | Liver | |
| --- | --- | --- | --- | --- | --- | --- |
|  | D_2%_ | D_mean_ | D_2%_ | D_mean_ | D_2%_ | D_mean_ |
| Protons 250MeV | 15.014 | 1.496 | 10.545 | 1.589 | 49.571 | 9.466 |
| VHEE 200MeV | 14.847 (-1 %) | 1.608 (+7 %) | 6.862 (-35 %) | 1.767 (+11 %) | 49.736 (0 %) | 9.910 (+5 %) |
| VHEE 150MeV | 16.539 (+10 %) | 1.776 (+19 %) | 6.578 (-38 %) | 1.895 (+19 %) | 49.793 (0 %) | 10.614 (+12 %) |

|  | Portal vein | | Ribs | | Small bowel | |
| --- | --- | --- | --- | --- | --- | --- |
|  | D_2%_ | D_mean_ | D_2%_ | D_mean_ | D_2%_ | D_mean_ |
| Protons 250MeV | 34.045 | 13.457 | 50.234 | 11.927 | 14.949 | 2.114 |
| VHEE 200MeV | 38.798 (+14 %) | 12.757 (-5 %) | 50.228 (0 %) | 13.372 (+12 %) | 13.818 (-8 %) | 2.030 (-4 %) |
| VHEE 150MeV | 41.579 (+22 %) | 13.041 (-3 %) | 50.143 (0 %) | 14.663 (+23 %) | 12.499 (-16 %) | 1.901 (-10 %) |

|  | Stomach | | Vena cava | |
| --- | --- | --- | --- | --- |
|  | D_2%_ | D_mean_ | D_2%_ | D_mean_ |
| Protons 250MeV | 14.702 | 4.843 | 37.855 | 11.723 |
| VHEE 200MeV | 12.954 (-12 %) | 5.143 (+6 %) | 31.381 (-17 %) | 9.936 (-15 %) |
| VHEE 150MeV | 10.285 (-30 %) | 4.288 (-11 %) | 31.173 (-18 %) | 9.589 (-18 %) |

Table S9: Plan metrics for the prostate case.

|  | Prostate PTV | | | | | |
| --- | --- | --- | --- | --- | --- | --- |
|  | CI50 | CI95 | HI98 | D2 | D95 | D98 |
| Protons 250MeV | 0.227 | 0.853 | 0.869 | 42.474 | 37.627 | 36.893 |
| VHEE 200MeV | 0.165 (-27 %) | 0.870 (+2 %) | 0.915 (+5 %) | 41.385 (-3 %) | 38.224 (+2 %) | 37.861 (+3 %) |
| VHEE 150MeV | 0.118 (-48 %) | 0.887 (+4 %) | 0.904 (+4 %) | 41.492 (-2 %) | 37.946 (+1 %) | 37.515 (+2 %) |

|  | Bladder | | L. femoral head | | R. femoral head | |
| --- | --- | --- | --- | --- | --- | --- |
|  | D2 | Dmean | D2 | Dmean | D2 | Dmean |
| Protons 250MeV | 35.778 | 2.992 | 3.410 | 0.223 | 0.813 | 0.053 |
| VHEE 200MeV | 36.845 (+3 %) | 4.919 (+64 %) | 6.086 (+78 %) | 0.995 (+346 %) | 3.706 (+356 %) | 0.477 (+800 %) |
| VHEE 150MeV | 36.533 (+2 %) | 5.414 (+81 %) | 5.349 (+57 %) | 1.124 (+404 %) | 3.542 (+336 %) | 0.611 (+1E3 %) |

|  | Penile bulb | | Rectal wall | | Rectum | |
| --- | --- | --- | --- | --- | --- | --- |
|  | D2 | Dmean | D2 | Dmean | D2 | Dmean |
| Protons 250MeV | 16.249 | 2.495 | 37.190 | 11.136 | 37.207 | 11.120 |
| VHEE 200MeV | 21.561 (+33 %) | 6.453 (+159 %) | 36.965 (-1 %) | 12.201 (+10 %) | 36.982 (-1 %) | 12.185 (+10 %) |
| VHEE 150MeV | 21.858 (+35 %) | 7.591 (+204 %) | 36.758 (-1 %) | 12.855 (+15 %) | 36.776 (-1 %) | 12.838 (+15 %) |

|  | Skin | |
| --- | --- | --- |
|  | D2 | Dmean |
| Protons 250MeV | 15.885 | 1.434 |
| VHEE 200MeV | 18.604 (+17 %) | 1.738 (+21 %) |
| VHEE 150MeV | 20.844 (+31 %) | 1.800 (+26 %) |
